# A tissue injury sensing and repair pathway distinct from host pathogen defense

**DOI:** 10.1016/j.cell.2023.03.031

**Published:** 2023-04-24

**Authors:** Siqi Liu, Yun Ha Hur, Xin Cai, Qian Cong, Yihao Yang, Chiwei Xu, Angelina M. Bilate, Kevin Andrew Uy Gonzales, S. Martina Parigi, Christopher J. Cowley, Brian Hurwitz, Ji-Dung Luo, Tiffany Tseng, Shiri Gur-Cohen, Megan Sribour, Tatiana Omelchenko, John Levorse, Hilda Amalia Pasolli, Craig B. Thompson, Daniel Mucida, Elaine Fuchs

**Affiliations:** 1 Robin Chemers Neustein Laboratory of Mammalian Development and Cell Biology, Howard Hughes Medical Institute, The Rockefeller University, New York, NY 10065, USA; 2 Cancer Biology and Genetics Program, Memorial Sloan Kettering Cancer Center, New York, NY 10065, USA; 3 McDermott Center for Human Growth and Development, Department of Biophysics, and Harold C. Simmons Comprehensive Cancer Center, University of Texas Southwestern Medical Center, Dallas, TX 75390, USA; 4 Laboratory of Mucosal Immunology, Howard Hughes Medical Institute, The Rockefeller University, New York, NY 10065, USA; 5 Bioinformatics Resource Center, The Rockefeller University, New York, NY 10065, USA; 6 Electron Microscopy Resource Center, The Rockefeller University, New York, NY 10065, USA; 7 These authors contributed equally; 8 Lead contact

## Abstract

Pathogen infection and tissue injury are universal insults that disrupt homeostasis. Innate immunity senses microbial infections and induces cytokines/chemokines to activate resistance mechanisms. Here, we show that, in contrast to most pathogen-induced cytokines, interleukin-24 (IL-24) is predominately induced by barrier epithelial progenitors after tissue injury and is independent of microbiome or adaptive immunity. Moreover, *Il24* ablation in mice impedes not only epidermal proliferation and re-epithelialization but also capillary and fibroblast regeneration within the dermal wound bed. Conversely, ectopic IL-24 induction in the homeostatic epidermis triggers global epithelial-mesenchymal tissue repair responses. Mechanistically, *Il24* expression depends upon both epithelial IL24-receptor/STAT3 signaling and hypoxia-stabilized HIF1α, which converge following injury to trigger autocrine and paracrine signaling involving IL-24-mediated receptor signaling and metabolic regulation. Thus, parallel to innate immune sensing of pathogens to resolve infections, epithelial stem cells sense injury signals to orchestrate IL-24-mediated tissue repair.

## In brief

Epithelial stem cells sense injury signals to activate an IL-24-mediated tissue repair pathway that is molecularly distinct but functionally parallel to pathogen-induced IFN signaling in innate immunity.

## Graphical Abstract

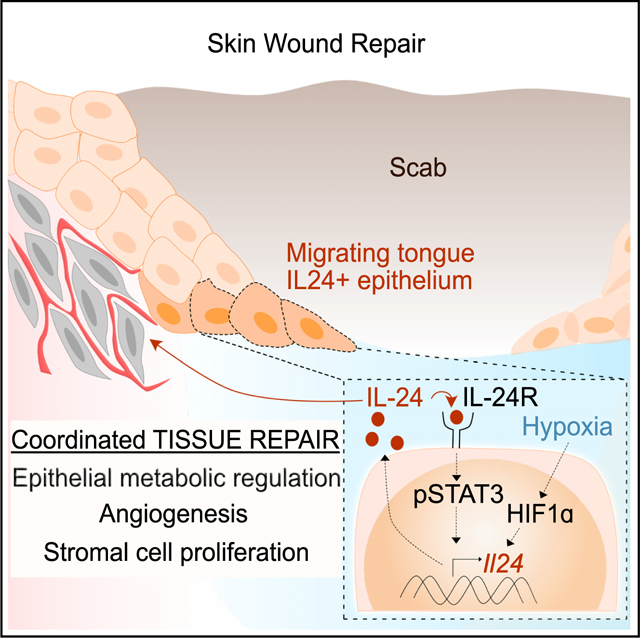

## INTRODUCTION

Maintaining homeostasis is a hallmark of biological systems, from unicellular organisms to mammals, and is exemplified by our ability to resolve disruptions, including pathogen infection and tissue injury.^1^ Barrier epithelial tissues of skin, lung, and intestine are the first line of defense against external assaults. Upon infection, these epithelia often sense pathogen-associated molecular pat- terns (PAMPs), such as “non-self” bacterial DNA or viral RNA, which activate pattern recognition receptors and downstream interferon response transcription factors (IRFs) to promote induction and secretion of type-I and -III interferons (IFNs).^2,3^ Upon IFN engagement, receptor Janus tyrosine kinases (JAKs) become activated, phosphorylating transcription factors STAT1/2 and orchestrating the cell-, tissue-, and organismal-level defense that resists and eliminates pathogens and restores homeostasis.^4^

**Figure 1.**
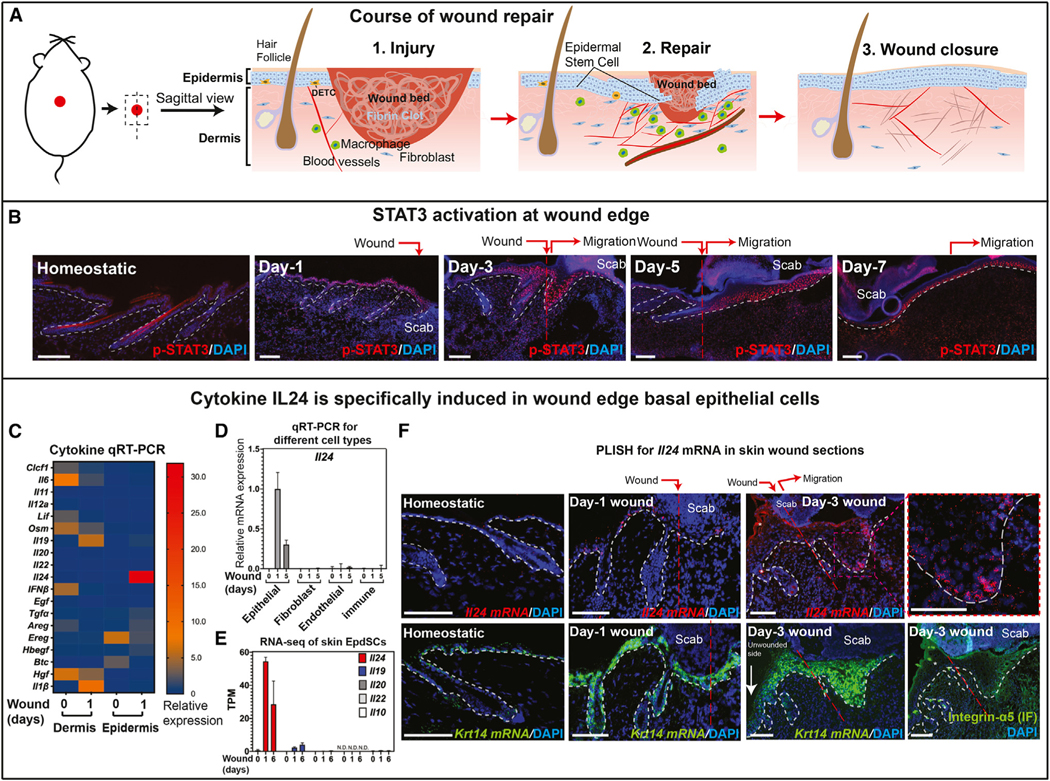
IL-24 is specifically produced by epithelial stem cells near the wound site (A) Schematic of the wound repair process in mouse skin. (B) Sagittal sections of homeostatic skin, and wounds (days indicated) immunolabeled for p-STAT3 at Tyr705 (n = 5 mice). (C) qRT-PCR for putative STAT3-targeting cytokines in homeostatic skin and day-1 wound. Il1β served as a positive control.^6^ Values were normalized to *Ppib* (n = 3 mice). (D) qRT-PCR of *Il24* mRNA in FACS-purified cell populations isolated from homeostatic and wounded skin (n = 3 mice). (E) IL-10 cytokine family expression from RNA-seq performed on FACS-purified EpdSCs from homeostatic and wounded skin. TPM, transcripts per kilobase million (n = 3 mice). (F) PLISH (proximity-ligation-based *in situ* hybridization) images of sagittal sections of homeostatic and wounded skin, probed for *Il24* and *Krt14* mRNA. Serial skin sections of *Il24* PLISH and immunolabeling of integrin-α5 in day-3 wounds. The red-boxed region was magnified and shown at the right to highlight the *Il24* PLISH signal in the re-epithelializing (migrating) epidermis. Asterisk (*) denotes autofluorescence of hair shaft and stratum corneum (n = 3 mice). Experiments were performed R ≥3×. White dotted lines, epidermal-dermal border; wound site, red dotted line; epidermal migration direction, red arrow. DAPI, nuclei; scale bars, 100 μm. Data in (D) and (E) are presented as mean ± SEM. N.D., not detected. See also Figure S1 and Tables S1 and S2.

Injury is another acute tissue-level insult that multicellular organisms must confront and respond to.^1,5^ Following injury, hemostasis initiates eschar (scab) formation, while neutrophils and macrophages enter damaged tissue to launch inflammation and clear debris (Figure 1A). Skin heals through re-epithelialization and dermal remodeling. This includes the tightly coordinated migration of epidermal progenitors (epidermal stem cells [EpdSCs]),^7–11^ followed by proliferation and regeneration of both epidermal and dermal components to restore skin organ homeostasis (Figure 1A).^5,12–16^

The molecular details underlying the complex wound repair process are still unfolding. Recent studies begin to reveal how tissue damage triggers immediate inflammatory responses.^17–20^ However, it remains poorly understood, especially in mammals, how injury is sensed by the host to coordinate progressive tissue-/organ-level repair. As a consequence, it is still largely unknown whether responses to tissue damage resemble the innate immune response to infection and, if so, how.

Exposed at the body surface, skin is ideal to interrogate how hosts sense and respond to tissue damage (Figure 1A). Here, we identify a wound-induced signaling pathway that can be triggered independently of microbes or adaptive immunity. We show that it is molecularly distinct but functionally similar to pathogen-induced IFN signaling in innate immunity. In this pathway, EpdSCs within the innermost (basal) layer at the wound edge sense wound-induced hypoxia as a damage signal to induce activation and signaling of IFN homolog interleukin-24 (IL-24). Despite being linked previously to injury,^21–24^ IL-24’s origins, mechanism of activation, and functions remain elusive. We now provide compelling evidence that in hypoxic conditions, an autocrine IL-24/IL-24-receptor signaling/STAT3 loop is induced, which then sustains the HIF1α-mediated expression of epidermal IL-24. In turn, IL-24 acts in an autocrine and paracrine fashion to coordinate re-epithelialization, re-vascularization, dermal fibroblast proliferation, and collagen deposition to restore the damaged tissue to homeostasis.

## RESULTS

### IL-24 is specifically expressed by EpdSCs near the wound site

Upon skin wounding, EpdSCs activate (phosphorylate) transcription factor STAT3 (p-STAT3), which is essential for their proliferation and migration at the wound edge.^13,25^ STAT3 is also activated in nearby dermal cells and remains high in both compartments until healing nears completion (~day-7; Figure 1B). The vital importance of STAT3 in tissue repair led us to wonder whether STAT3’s functional roles in tissue repair might be analogous to those played by STAT1/2 in pathogen resistance.^26^

To further probe this relation, we compiled a list of signaling factors reported to activate STAT3 (Table S1).^27–29^ To evaluate their early response to skin injury, we introduced a 6 mm full-thickness wound, and then at day-0 and day-1 post-injury, we microdissected an ~0.5-mm skin region surrounding the wound site and analyzed mRNAs from enzymatically separated dermal and epidermal fractions by quantitative reverse transcription polymerase chain reaction (qRT-PCR). Among factors capable of activating STAT3, only a few exhibited a wound-induced expression pattern. *Il24* stood out as a cytokine induced after injury and largely, if not exclusively, in the epidermal fraction (Figure 1C).

IL-24 is a conserved member of the IL-10 family, which includes IL-10, IL-22, IL-19, IL-20, and IL-24^29,30^ (Table S2). Unbiased phylogenetic analyses indicated that this family and its receptors^28^ share greater sequence/structure homology to IFN and IFN-receptors than other cytokines/cytokine-receptors (Figures S1A and S1B; Table S3). Notably, the heterodimeric receptor subunits of the IFN and IL-10 families also sub-clustered, suggestive of a common ancestral heterodimeric receptor specific to these two families (Figure S1B). In contrast to IFNs, however, the IL-10 cytokine family has not been as clearly linked to pathogens/danger signals. This raises the tantalizing possibility that, during evolution, these pathways may have bifurcated from a common ancestor to cope with the increasing diversity of pathogens and injuries.

Most studies on IL-24 center on cultured cells.^22–24,31^ IL-24’s expression, regulation, and functions in natural physiological settings remain elusive, with both positive and negative effects on wound repair described. To pinpoint the cells expressing *Il24* in skin wounds and assess IL-24’s possible importance in repair, we performed fluorescence-activated cell sorting (FACS) and purified the major cellular constituents in and within 0.5–1 mm of the wound bed at times during re-epithelialization (Figure S1C). *Il24* was induced primarily within the EpdSC fraction (integrin-α6^hi^SCA1^hi^CD34^neg^CD45^neg^ CD31^neg^PDGFR⍺^neg^ CD117^neg^) at the wound site (Figure 1D). Among other IL-10 family members, only *Il19* showed weak induction in EpdSCs following injury (Figure 1E).

Probing deeper, we performed 10x single-cell RNA sequencing (scRNA-seq) of skin wounds. *Il24* mRNA was predominantly within the epithelial cell cluster (*Krt14*^+^) co-expressing basal EpdSC marker integrin-α6 (*Itga6*) (Figure S1D; red arrows). Analysis of additional 10x scRNA-seq data on wounds^32^ was consistent with these findings.

We next combined immunofluorescence microscopy and proximity-ligation-based fluorescent *in situ* hybridization (PLISH)^33^ to localize *Il24*. While *Krt14* PLISH marked the epidermis of both homeostatic and wound-induced tissue, *Il24* PLISH was only detected following injury, where it appeared within 24 h in EpdSCs near the wound site (Figure 1F). As wound-edge EpdSCs migrate into the wound bed, they induce integrin-α5^+^.^7,10^ By day-3, the *Il24* PLISH signal had intensified within integrin-$\text{α}5^{+}$ basal EpdSCs of the re-epithelializing tongue (Figure 1F). This finding corroborated both our bulk RNA-seq and qPCR results of *Il24* mRNA enrichment in the integrin-$\text{α}5^{+}$ migrating EpdSCs (Figures S1E and S1F). Together, these data pointed to the view that an as yet undetermined injury signal(s) is received by nearby EpdSCs, causing them to produce IL-24 predominantly at the migrating epidermal front of the wound bed.

### Injury-induced IL-24 signaling resembles infection-induced IFN innate immune signaling

**Figure 2.**
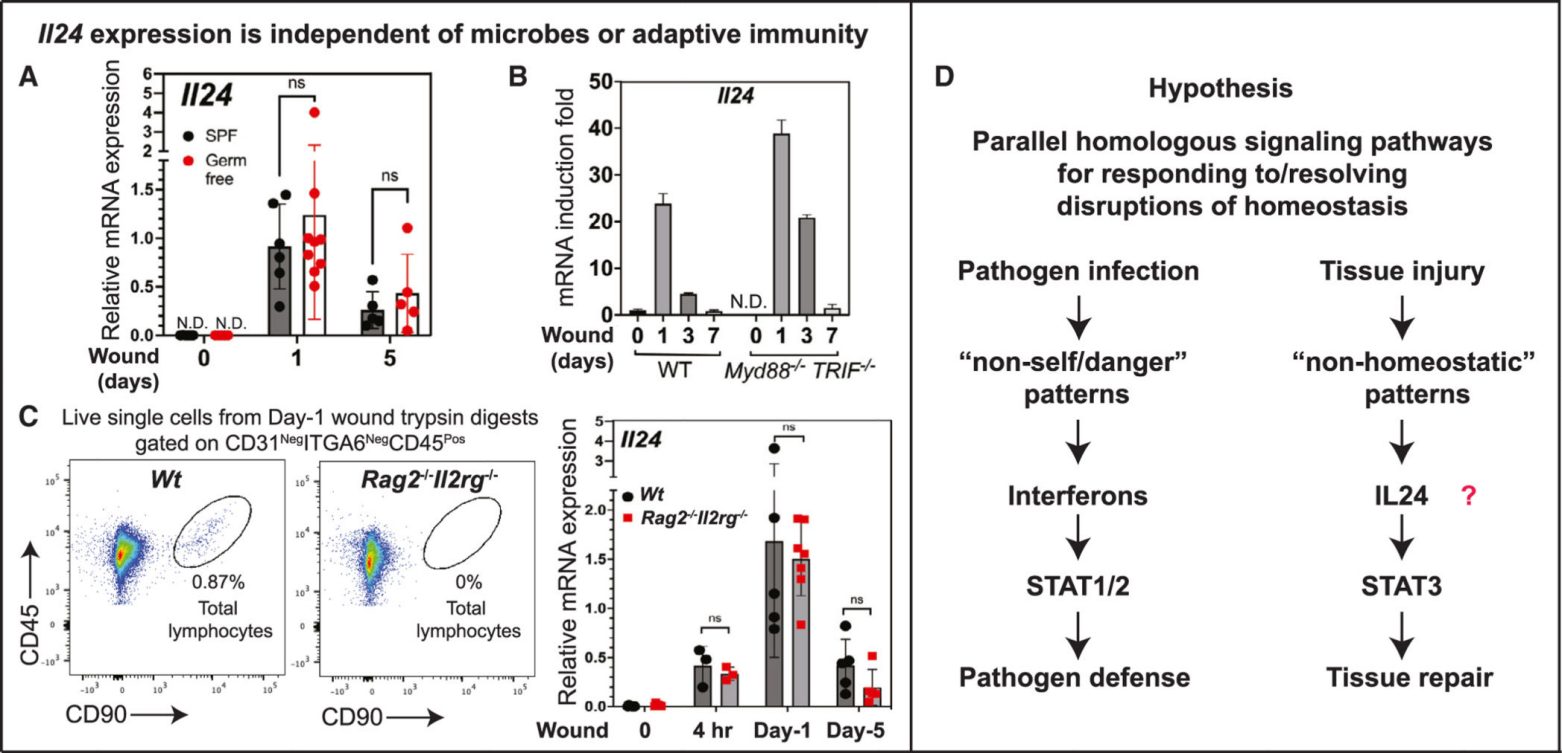
Injury-induced IL-24 signaling resembles infection-induced interferon signaling (A) qRT-PCR of *Il24* mRNA in FACS-purified EpdSCs from homeostatic and wounded skin from specific-pathogen-free (SPF) vs. germ-free (GF) C57BL/6J WT mice (SPF, n = 5–6, GF, n = 5–9 mice). (B) qRT-PCR of *Il24* mRNA in epidermis microdissected from homeostatic and wounded skin from WT vs. *Myd88*^−/−^*Trif*^−/−^ mice (n = 3 mice per genotype; representative of 3 independent experiments). (C) qRT-PCR analysis of *Il24* mRNA in EpdSCs FACS-purified from homeostatic and wounded skin from WT vs. *Rag2/IL2rg* DKO mice. Note that *Rag2/IL2rg* DKO mice lack all functional lymphocytes (n = 5–7 mice per genotype). (D) Diagram depicting our central hypothesis that parallel but distinct signaling pathways are used for responding to and resolving pathogen infection and tissue injury. Steps tackled in current study are highlighted by question marks. Data in (A)–(C) are presented as mean ± SEM. Statistical significance was determined using two-tailed unpaired Student’s t tests; ns; not significant; N.D.; not detected. Dots in the graphs indicate data from individual mice. See also Table S3.

During infection, pathogen-derived signals trigger a host innate immune response, which frequently leads to IFN production and pathogen clearance.^2^ Given that in wounds EpdSCs are exposed to microbes, we first tested whether commensal bacteria/microbes at the skin surface are responsible for inducing *Il24* following injury. Intriguingly, mice raised under completely sterile (germ-free) conditions still robustly induced *Il24* in EpdSCs at the wound edge (Figure 2A). Consistently, *Il24* was also induced following wounding of *Myd88*^−/−^*Trif*^−/−^ mice, which lack Tolllike receptor (TLR) signaling essential for many microbial responses (Figure 2B). This was notable, as TLR-signaling functions in the production of some other IL-10 family members.^34,35^ Together, these results provided compelling evidence that distinct from pathogens/danger signals, which trigger type-I IFNs, a microbe-independent tissue damage signal induces *Il24* at the wound edge.

Type-I IFNs are induced by the activation of innate immune pathways, whereas type-II IFN (IFN-γ) is predominantly induced by lymphocytes.^36^ Recent studies show that adaptive immune cells involving regulatory T cells and IL-17A-expressing Rorγt^+^ T cells are important for wound repair.^11,37^ We thus examined whether the adaptive immune system might be responsible for inducing IL-24 in wound-edge EpdSCs. However, when compared against wild-type (WT) mice, *Rag2/Il2rg* double knockout (DKO) mice, which lack functional lymphocytes alto- gether,^38,39^ still temporally induced *Il24* in EpdSCs at the wound site (Figure 2C). These data point to an upstream damage signal(s) that induces *Il24* at the wound site and is independent of adaptive immune cells.

Based upon our collective evidence, we hypothesized that, analogous to the sensing of pathogen-derived non-self patterns that prompt somatic cells to activate type-I IFN-receptor-STAT1/2 signaling in defense against microbial infections, injury-induced signals that do not exist in homeostatic conditions (“non-homeostatic” patterns) may be sensed directly by EpdSCs at the wound edge to trigger the activation of IL-24-receptor-p-STAT3 signaling and initiate tissue-damage-mediated repair (Figure 2D).

### STAT3 activation and epithelial proliferation rely upon IL-24 in wound repair

**Figure 3.**
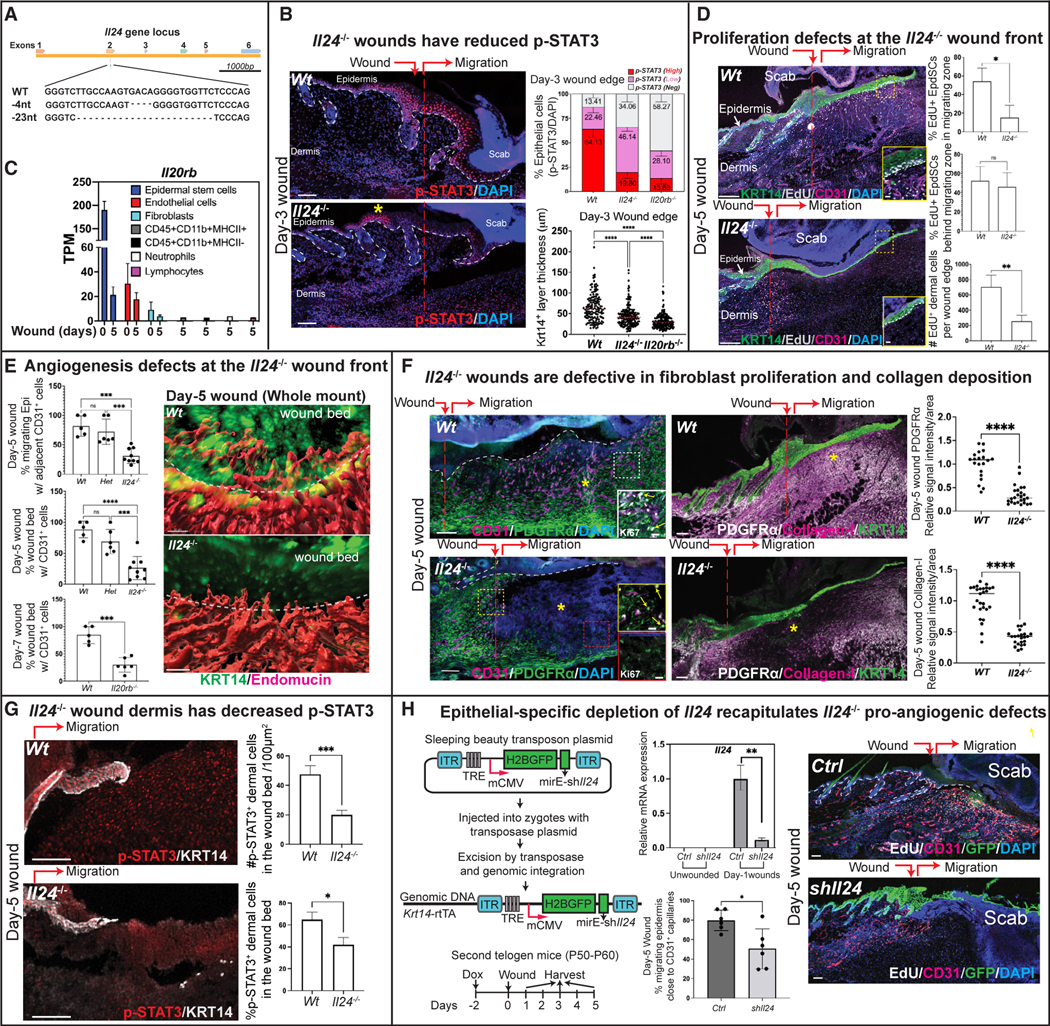
Epithelial-expressed IL-24 coordinates dermal repair and re-epithelialization (A) Schematic of two C57BL/6J *Il24*^−/−^ mouse strains generated by CRISPR-Cas9-mediated frameshift deletions within *Il24* exon 2. Impairments of wound repair were indistinguishable between two loss-of-*Il24-*function strains, used interchangeably for experiments. (B) Sagittal sections of day-3 wounds from wild-type (WT) vs. *Il24* null mice immunolabeled for p-STAT3. Note that p-STAT3 is still seen in *Il24* null wounded epidermis (asterisk). Graphs show quantifications of the percentage of EpdSCs expressing p-STAT3 (upper), and the thickness of keratin 14 (KRT14^+^) progenitor layers (lower) (n = 5 mice per genotype). (C) *Il20rb* RNA-seq of FACS-purified cell populations from homeostatic skin and day-5 wounds (note: immune cells were only from day-5 wounds). TPM, transcripts per kilobase million (n = 5 mice). (D) Sagittal sections of day-5 wounds immunolabeled for KRT14 (epidermis), CD31 (endothelial cells), and labeled with 5-ethynyl-2′-deoxyuridine (EdU) (proliferation). Boxed regions are magnified in insets to better visualize EdU incorporation of S-phase cells (scale bars, 10 μm). Graphs show quantifications of percentage of EdU^+^ cells in epidermis and dermis. For epidermis, quantifications were performed separately for the cells in the migrating zone (to the right of the wound site) and behind the migrating zone (to the left of the wound site) (n = 5 mice per genotype). (E) Left: quantifications of the percentages of migrating epidermis displaying adjacent CD31^+^ endothelial cells (top) and the percentages of the wound beds at day-5 and −7 post wounding that were repopulated with sprouting blood vessels (CD31^+^ cells) (middle and bottom). Mouse genotypes are as indicated (see STAR Methods). Top and middle: WT: n = 5, *Il24* Het: n = 6, *Il24*^−/−^: n = 9 mice, one-way ANOVA, Tukey’s multiple comparisons test; bottom, WT: n = 5, *Il20rb*^−/−^:n =6 mice, two-tailed unpaired t test; dots in the graphs indicate data from individual mice. Right: Images of whole-mount immunofluorescence microscopy and 3D image reconstruction performed on day-5 wounds from WT vs. *Il24* null mice (scale bars, 50 μm. Immunolabeling was for KRT14 [epidermis] and endomucin [blood vessels]) (n = 3 mice per genotype). (F) Sagittal sections of day-5 wounds immunolabeled for CD31 and PDGFRα (left), or for PDGFRα, collagen-I, and KRT14 (right). Asterisk (*) denotes a paucity of fibroblasts (${\text{PDGFRα}}^{+}$) and their deposition of collagen-I ECM in the dermis of *Il24*^−/−^ skin. The boxed region magnified in the color-coded insets shows additional Ki67 immunolabeling (Scale bars, 20 μm). Yellow arrows denote Ki67^+^ proliferating fibroblasts (Ki67^+^PDGFR^+^). Quantifications are of fibroblast amount (PDGFRα intensity, upper) and collagen deposition (lower) (n = 5 per genotype). (G) Sagittal sections of day-5 wounds immunolabeled for p-STAT3 and KRT14. Percentage and number/area of p-STAT3^+^ dermal cells beneath the wound bed are quantified (n = 3 mice per genotype). (H) Left: sleeping beauty system used to generate epidermal-specific *Il24* mRNA knockdown mice. Middle top: qRT-PCR of *Il24* mRNA in FACS-purified EpdSCs from homeostatic and day-1 wounded skins from control (Ctrl) vs. sh*Il24* mice (n = 5–6 mice for each genotype). Right: sagittal sections of day-5 wounds from control (Ctrl) vs. sh*Il24* mice immunolabeled for CD31, KRT14 and labeled with EdU. Percentage of migrating epidermis adjacent to CD31^+^ capillaries is quantified in middle bottom panel (n = 6 mice per genotype). White dotted lines: epidermal-dermal border; wound site, red dotted line; epidermal migration direction, red arrow. DAPI, nuclei; scale bars except for boxed regions and whole mount: 100 μm. Data in (B)–(H) are presented as mean ± SEM. Dots in the graphs (E) and (H) indicate data from individual mice. Statistical significance was determined using two-tailed unpaired Student’s t tests in (D), (E; bottom panel), (F), (G), and (H); and using one-way ANOVA, Tukey’s multiple comparisons test in (B) and (E; top two panels); **** p < 0.0001; *** p < 0.001; ** p < 0.01; * p < 0.05; and ns, not significant. See also Figures S2–S5.

If this IL-24-mediated tissue injury response is analogous to pathogen infection where IFNs are upstream of STAT1/2, then IL-24 should be important for STAT3 activation in wounds. To test this hypothesis and further interrogate the physiological significance of IL-24 in wound repair, we engineered *Il24*^−/−^ mice by directly injecting *Il24* guide RNA and CAS9 protein into fertilized embryos. Two independent CRISPR-Cas9-generated *Il24*^−/−^ lines were generated that harbored loss-of-function frameshift mutations within exon 2 (Figures 3A and S2A).

Adult *Il24*^−/−^ mice were healthy, fertile, and indistinguishable from WT littermates at baseline. Upon challenge, however, the wounded *Il24*^−/−^ epidermis displayed a markedly reduced ability to activate STAT3 specifically near the wound edge where IL-24 was normally expressed (Figures 3B and S2B). In marked contrast, despite IL-6 being oft-considered the major STAT3-activating cytokine in skin,^40^ *Il6* ablation showed little effect on p-STAT3 in wound-induced skin (Figure S2C). Further consistent with reduced p-STAT3 in the *Il24*^−/−^ migrating epithelial tongue, the thickness of KRT14^+^ progenitor layers at the wound edge was markedly reduced compared with WT wounded skin (Figure 3B). These data highlight parallels between pathogen and damage response pathways and suggest that IL-24 acts directly on the wound-edge epithelium to sustain p-STAT3 and promote repair.

Deletion of IL-20RB, the pan subunit for IL-24-receptor signaling, also displayed defects in p-STAT3 and re-epithelialization, setting IL-24 apart from IL-22 and IL-10, which have been implicated in wound repair but use different heterodimeric receptors.^41,42^ However, the response to IL-20RB loss was even more robust than IL-24 alone (Figures 3B, S2D, and S2E). This accentuated phenotype is likely attributable to redundancy with IL-19, which is the only other IL-20 subfamily member that both utilizes IL-24 receptors^43^ and was wound-induced, albeit at lower levels than *Il24* (Figures 1C and 1E). RNA-seq analysis confirmed that the shared IL-24/IL-19 receptor subunit IL-20RB, as well as the other two co-receptors, was highly expressed in EpdSCs, indicative of the importance of epithelial IL-24/IL-19 signaling in STAT3 activation and wound re-epithelialization (Figures 3C and S2F).

### Epithelial IL-24 coordinates dermal repair and re-epithelialization

The robust epidermal expression of both IL-24 and its receptor was consistent with autocrine IL-24 action, as discussed above. Interestingly, however, despite lower IL-24-receptor expression in mesenchymal cells, *Il24*^−/−^ wounded skin dermis displayed marked proliferation defects (Figures 3D and S3A). Seeking the source of these dermal defects, we first co-immunolabeled for markers of proliferation and endothelial cells (CD31, endomucin), where IL-24-receptor expression was appreciable. Notably, in the absence of IL-24, a striking impairment arose in the sprouting of regenerating blood capillaries that normally account for ~50% of proliferating dermal cells in day-5 post-injured skin (Figures S3B–S3D). Consistently, a recently developed clearing method^44^ in conjunction with whole-mount immunofluorescence and 3D image reconstruction of day-5 wounded skin revealed a marked paucity of dermal blood vessel angiogenesis, which normally closely associates with the overlying migrating epithelial tongue (Figures 3E and S3E). 70% of the epidermis that migrated into the *Il24*^−/−^ wound bed lacked underlying vascular support, without which epidermal proliferation plummeted (Figures 3D and 3E). Consistent with the importance of IL-24-receptor signaling, *Il20rb*^−/−^ mice exhibited a similar paucity of proliferating blood vessels migrating into the wound bed, a defect still evident even at day-7 after wounding (Figures 3E and S3F).

The remaining proliferating dermal cells in WT day-5 wounds were mostly PDGFRα^+^ fibroblasts, but these too were largely absent in the *Il24*^−/−^ day-5 wound bed (Figures 3F and S3C). Consistently, the *Il24*^−/−^ wound bed displayed a paucity of type-I collagen, an essential extracellular matrix (ECM) component secreted by mature fibroblasts to provide structural support for vasculature and the overlying epidermis.

Although IL-24 induction did not require pathogens nor adaptive immunity, innate immune cells are involved in tissue damage responses, and hence we examined whether they responded to IL-24 loss. Consistent with their paucity of IL-24 receptors (Figures 3C and S2F), innate immune cell numbers were largely insensitive to IL-24 status (Figure S4A). Besides neutrophils, macrophages were the most abundant immune cells in the wound bed. Although their total cellularity was similar, macrophage distribution and maturation were noticeably perturbed in *Il24*^−/−^ wounded skin.

In day-5 wounds, *Arg1*^+^ and MHCII (*H2-aa*)^+^ cells were the two major subpopulations of macrophages/monocytes (Figure S4B). In WT wounds, ARG1^+^ macrophages appeared underneath the migrating epithelial tongue by day-3, and by day-5, as dermal proliferation and angiogenesis populated the region, ARG1^+^ cells retreated deeper into the wound bed where re-epithelialization and angiogenesis had not yet taken place. In striking contrast, ARG1^+^ cells in *Il24*^−/−^ day-5 wounds persisted underneath the migrating epithelium and erroneously overlapped with dermal proliferating cells (Figure S4C).

Additional perturbations were noted in MHCII^+^ cells, which normally tracked with proliferating dermal cells migrating into the wound bed. In the *Il24*^−/−^ wound bed, they failed to do so (Figure S4C, middle). Given that MHCII^+^ and ARG1^+^ macrophages strongly expressed *Vegfa* (Figure S4B), they likely both contribute to angiogenesis, providing an avenue for why angiogenesis may have been altered in wounds of our IL-24-deficient mice. Indeed, VEGFA immunofluorescence was considerably stronger in the dermal wound bed of WT versus *Il24*^−/−^ mice (Figure S4C, right). Thus, despite not responding directly, macrophages were nonetheless sensitive to IL-24-dependent changes in the wound bed. Given the known impact of fibroblasts on macrophages,^45^ the paucity of fibroblasts in the *Il24-*deficient wound bed may further contribute indirectly to these perturbations.

Toluidine blue staining of semithin tissue sections and transmission electron microscopy further substantiated these defects in restoring dermal cellularity (Figures S5A–S5C). The paucity of both mature dermal fibroblasts and abundant collagen deposition, coupled with the persistence of fibrin clots (pseudo-colored in green), left the migrating *Il24*^−/−^ epithelial tongue atop a fibrin clot rather than collagen-based ECM. The failure to efficiently clear dermal fibrin and cell debris, including red blood cells (RBCs), further underscored the disorganization of macro- phages. These findings underscored an overall decoupling of the normal repair process.

Visually, in comparison with WT, the *Il24*^−/−^ wound healing course was delayed by ~4 days, while hair re-growth, which relies upon proper epithelial-mesenchymal signaling, exhibited delays of up to 2 weeks post-injury, also seen at the histological level (Figures S5D and S5E). Wounds eventually healed and hairs regrew. This did not appear to involve obvious compensatory action, as later stage induction of other IL-20 family members—other than a transient increase of *Il19*—was not observed (Figure S5F). Rather, the results further reflected the dispensability of IL-24 for skin homeostasis.

### Epithelial-specific depletion of IL-24 recapitulates proangiogenic defects in *Il24*^−/−^ wounds

Although our data showed that, in skin, IL-24 is predominantly produced by wound-edge epithelial cells, IL-24 had previously been reported in other cell types and tissues.^46–50^ The broad range of wound-related defects upon whole body loss of IL-24 function coupled with a general decline in p-STAT3 signal within the wound bed (Figure 3G) mandated the need to know whether these defects originated specifically from the inability to induce IL-24 in the skin epithelium following injury. To this end, we generated inducible, skin-epithelium-specific *Il24-*mRNA knockdown mice by directly injecting *Krt14-rtTA*fertilized mouse eggs with a sleeping beauty system, including two plasmids encoding (1) transposase and (2) transposable elements, including H2BGFP, followed by sh*Il24* (miRE-sh*Il24*) driven by a TRE regulatory element activatable by the doxycycline (Dox)-sensitive transactivator rtTA (Figure 3H).

The majority of skin epithelial progenitors of both founder and F1 offspring mice efficiently and stably integrated the transposon, as indicated by H2BGFP in >90% of epidermal cells following Dox administration. In these sh*Il24* animals, Dox also efficiently silenced wound-induced *Il24* mRNA. Importantly, and as we had observed with full-body *Il24*^−/−^ wounds, epidermal-specific *shIl24* wounds failed to properly coordinate re-epithelialization and dermal angiogenesis (Figure 3H).

The expression of IL-24 receptors by endothelial cells and fibroblasts suggested that wound-induced epidermal IL-24 was triggering paracrine effects (Figures 3C and S2F). The paucity of p-STAT3 in both dermis and epidermis of *Il24*^−/−^ skin added fuel to this fire (Figure 3G). Indeed, upon treating primary endothelial and fibroblast cultures with recombinant IL-24, we observed robust p-STAT3 activation and cell proliferation (Figure S3G).

### Ectopic IL-24 induction in homeostatic skin epithelium elicits a wound-like response in the absence of injury

**Figure 4.**
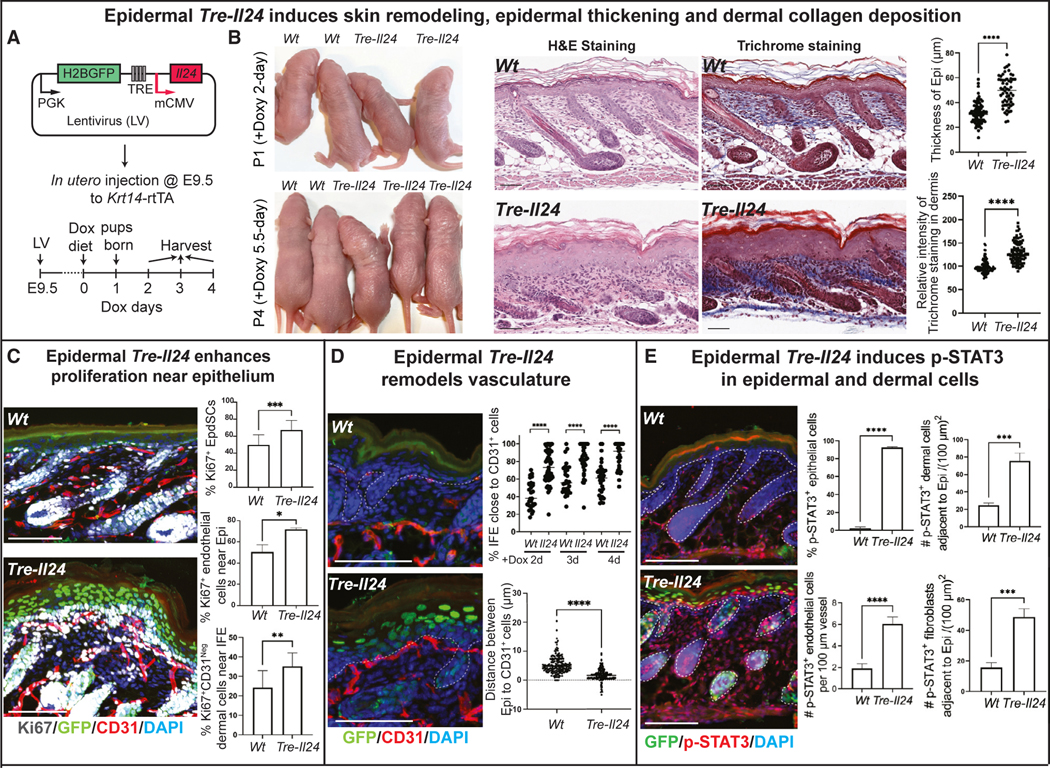
Ectopic IL-24 induction in homeostatic skin epithelium elicits a wound-like response without injury (A) Schematic of the generation of TRE-IL-24 mice. Selective targeting to skin EpdSCs was achieved by packaging the transgene in a lentivirus and *in utero* injection into the amniotic sacs of E9.5 mouse embryos genetic for the *Krt14-rtTA* doxycycline inducible transcriptional activator. The lentivirus also contained a constitutively expressed *Pgk-H2BGFP* to monitor integration efficiency. Skins were harvested after mice were fed Dox food for 2, 3, or 4 days. (B) Left: images of mice at postnatal days 1 and 4. Note flaky skin phenotype, evident by day-4. Right: Images of hematoxylin and eosin (H&E) staining and trichrome staining performed on sagittal sections of homeostatic skins from Dox-fed WT and *Tre-Il24* mice. Quantifications are of epidermal thickness and intensity of trichrome staining to evaluate dermal collagen deposition (n = 3 mice per genotype). (C) Sagittal sections of homeostatic skins from WT and *Tre-Il24* mice immunolabeled for Ki67, GFP and CD31. Quantifications are of percentages of proliferating (Ki67^+^) EpdSCs (top), and underlying endothelial cells (Ki67^+^CD31^+^) (middle) and non-endothelial dermal cells (Ki67^+^CD31^−^) (bottom) (n = 3 mice per genotype). (D) Sagittal sections of homeostatic skins from WT and *Tre-Il24* mice immunolabeled for GFP and CD31. Quantifications are of percentage of interfollicular epidermis close to CD31^+^ endothelial cells (top), and the distance (mm) between epidermis and CD31^+^ vasculature (bottom) (n = 3 mice per genotype per time point). (E) Sagittal sections of homeostatic skins from WT and *Tre-Il24* mice were immunolabeled for GFP and p-STAT3. Prior to collecting skins, mice were given Dox food for 2 days. Quantifications are of percentage of p-STAT3^+^ epidermal, endothelial, and fibroblast cells. Quantifications of dermal cell types were made by performing similar immunofluorescence as for epidermis, but using antibodies against CD31 and PDGFα, respectively (n = 3 mice per genotype). White dotted lines: epidermal-dermal border. DAPI, nuclei; scale bars, 100 μm. Data in (B)–(E) are presented as mean ± SEM. Experiments were performed R ≥3×. Statistical significance was determined using two-tailed unpaired Student’s t tests; **** p < 0.0001; *** p < 0.001; ** p < 0.01; and * p < 0.05.

As IL-24 is specifically activated following injury, we asked whether its ectopic activation might be sufficient to elicit a wound-like response in the absence of injury. A prior study in which IL-24 was constitutively ectopically expressed in skin, starting in embryogenesis, led to epidermal hyperplasia but also neonatal lethality,^51^ emphasizing the necessity of an inducible approach to unravel the deeper complexities underlying IL-24’s actions. Using our powerful *in utero* lentiviral delivery method,^52^ we transduced the skin of mice genetic for an EpdSC (*Krt14*) specific, Dox-inducible rtTA with *Il24* driven by an rtTA-regulated enhancer (TRE) (Figure 4A).

Within 48 h of Dox-induction, radical changes arose, marked by enhanced epidermal thickness, elevated dermal collagen deposition, and local vascular remodeling directly beneath the EpdSC layer (Figures 4B–4D). These features were accompanied by marked increases in epidermal and dermal proliferation and, a few days thereafter, overt gross phenotypic features of a hyperproliferative skin state appeared (Figures 4B and 4C).

In wounded WT skin, the strongest p-STAT3 signal was in epidermal cells, which also expressed the highest level of IL-24 and IL-24 receptors, suggestive of autocrine signaling (Figure 4E). Despite lower levels of IL-24 receptors, endothelial cells and fibroblasts also displayed p-STAT3 in induced IL-24 skin (Figure 4E). Thus, even in the absence of injury, epidermal-specific IL-24 induction was sufficient to elicit a tissue-level wound-like response with both autocrine (epidermal) and paracrine (dermal) IL-24-receptor activation.

### Tissue-damage-associated hypoxia and HIF1α in wounds are important for robust *Il24* expression

We next searched for upstream signals that lead to *Il24* induction. Our data thus far indicated that the injury signal(s) must be a non-homeostatic pattern that is independent of microbes or adaptive immune cells and only unleashed after wounding. Further corroborating this point, this signal was independent of TNF signaling (Figure S6A), indicating that the mechanism that induces *Il24* in a physiological wound is distinct from the patho- logical scenario where the inhibitor of nuclear factor kappa-B kinase subunit beta (IKKβ) is deleted from skin.^53^

**Figure 5.**
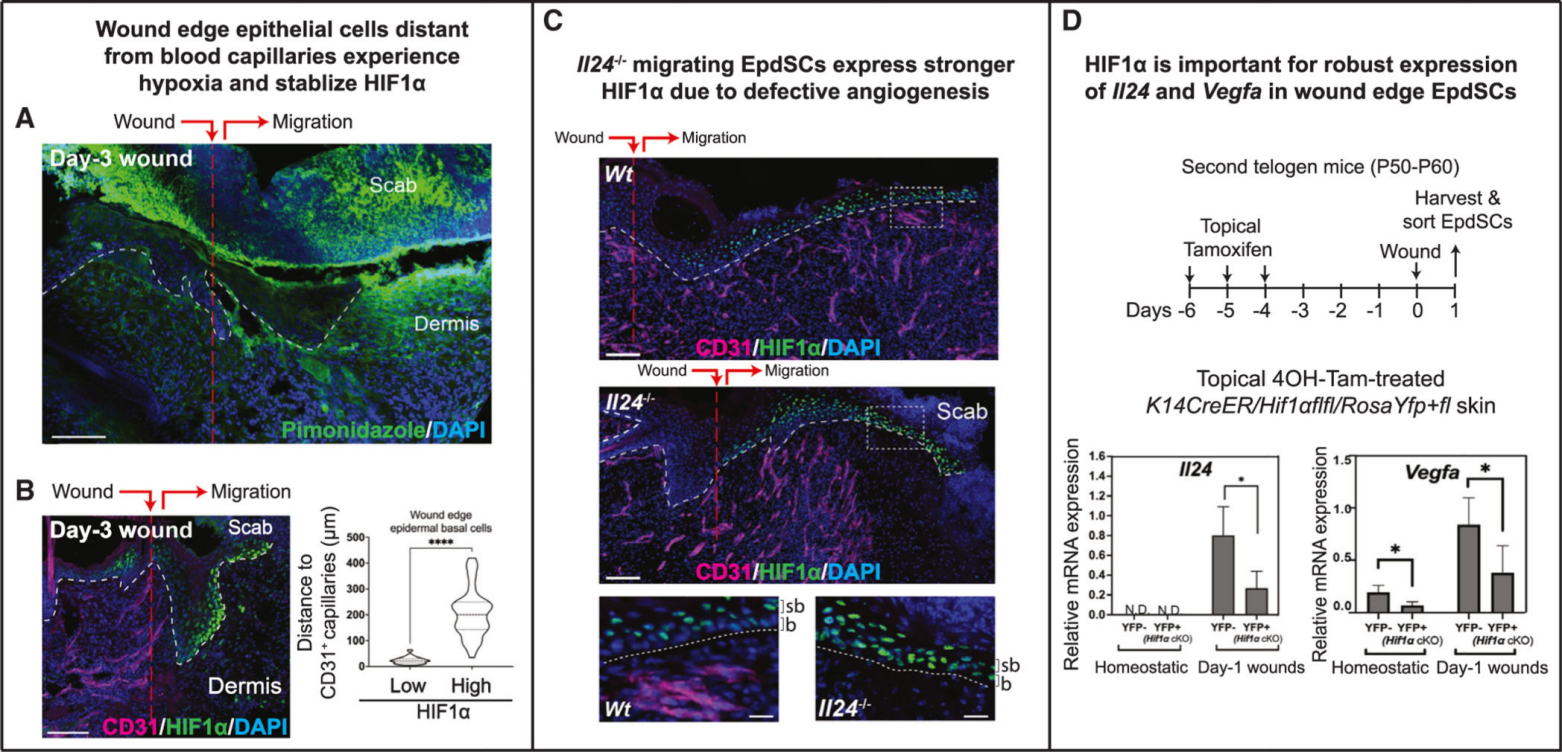
Tissue-damage-associated hypoxia and HIF1α in wounds are important for robust *Il24* expression (A) Sagittal section of day-3 wound harvested just after pimonidazole injection to label tissue hypoxia (n = 5 mice). (B) Sagittal section of day-3 wound immunolabeled for CD31 and HIF1α. The distance (μm) from ${\text{HIF1α}}^{\text{Low}}$ vs. ${\text{HIF1α}}^{\text{High}}$ EpdSCs to the nearest CD31^+^ blood vessels is quantified (n = 5 mice). (C) Sagittal sections of day-5 wounds from WT and *Il24* null mice immunolabeled for CD31 and HIF1α. Boxed regions of the migrating epidermal tongue are magnified at right (scale bars, 20 μm). b, basal EpdSCs; sb, suprabasal epidermal cells (n = 5 mice per genotype). (D) Schematic of the experiment and qRT-PCR of *Il24* and *Vegfa* mRNA in YFP^−^ (Hif1α WT) or YFP^+^ (Hif1α*Δ*exon2) FACS-purified EpdSCs from homeostatic skin and from day-1 wounds of *Krt14CreER*; ${\text{Hif1α}}^{\text{fl/fl}}$; *RosaYFP*^*+/fl*^ mice treated with topical 4OH-Tam (n = 5 mice). White dotted lines: epidermal-dermal border; wound site, red dotted line; epidermal migration direction, red arrow. DAPI, nuclei; scale bars except for the boxed regions: 100 μm. Data in (B) and (D) are presented as mean ± SEM. Experiments were performed R ≥ 3×. Statistical significance was determined using two-tailed unpaired Student’s t tests; **** p < 0.0001; * p < 0.05. See also Figures S6.

In WT mice, epidermal proliferation during wound repair paralleled newly sprouting blood capillaries (Figure S6B). In *Il24-*null mice, a deficiency in dermal angiogenesis following injury was among the most notable defects (Figures 3D and 3E). Hence, we posited that the non-homeostatic pattern(s) sensed by EpdSCs following injury may emanate from severed blood vessels. Turning to tissue hypoxia as a top candidate, we began by verifying that the early wound bed of WT skin is hypoxic.^54,55^ Indeed, hypoxia probe pimonidazole^56^ strongly labeled the wound bed and, correspondingly, hypoxia-stabilizing transcription factor HIF1α was nuclear, beginning at the immediate WT wound edge following injury and extending to the migrating (IL-24-expressing) epithelial tongue (Figures 5A, 5B, and S6C). Additionally, the intensity of nuclear HIF1α in EpdSCs correlated with distance from blood capillaries, with the most robust signal always in the epithelial tongue at least 100 μm ahead of regener- ating (day-3) blood capillaries.

In contrast to day-5 WT wounds, where HIF1α had waned in epidermis concomitant with newly sprouted underlying blood capillaries (Figure S6C), day-5 *Il24*^−/−^ wounds resembled that of WT day-3 wounds, displaying strong nuclear HIF1α in overlying epidermis that still lacked close contact with blood capillaries (Figure 5C). These data placed hypoxia and HIF1α upstream of IL-24.

If hypoxia regulates *Il24* expression, the loss of HIF1α might be expected to deleteriously affect wound-stimulated *Il24* induc- tion. Indeed, this was the case, as parallel to the well-established HIF1α target gene, *Vegfa, Il24* mRNA levels plummeted when HIF1α was conditionally ablated within epidermis prior to wounding (Figures 5D and S6D). Together with IL-24’s importance for dermal blood capillary regeneration and EpdSC proliferation, these results suggested that following EpdSC-sensing of wound-generated hypoxia, IL-24 was induced in order to promote revascularization and proper re-epithelialization.

### Critical roles for both hypoxia/HIF1α and IL-24-receptor/STAT3 signaling in governing robust *Il24* expression

**Figure 6.**
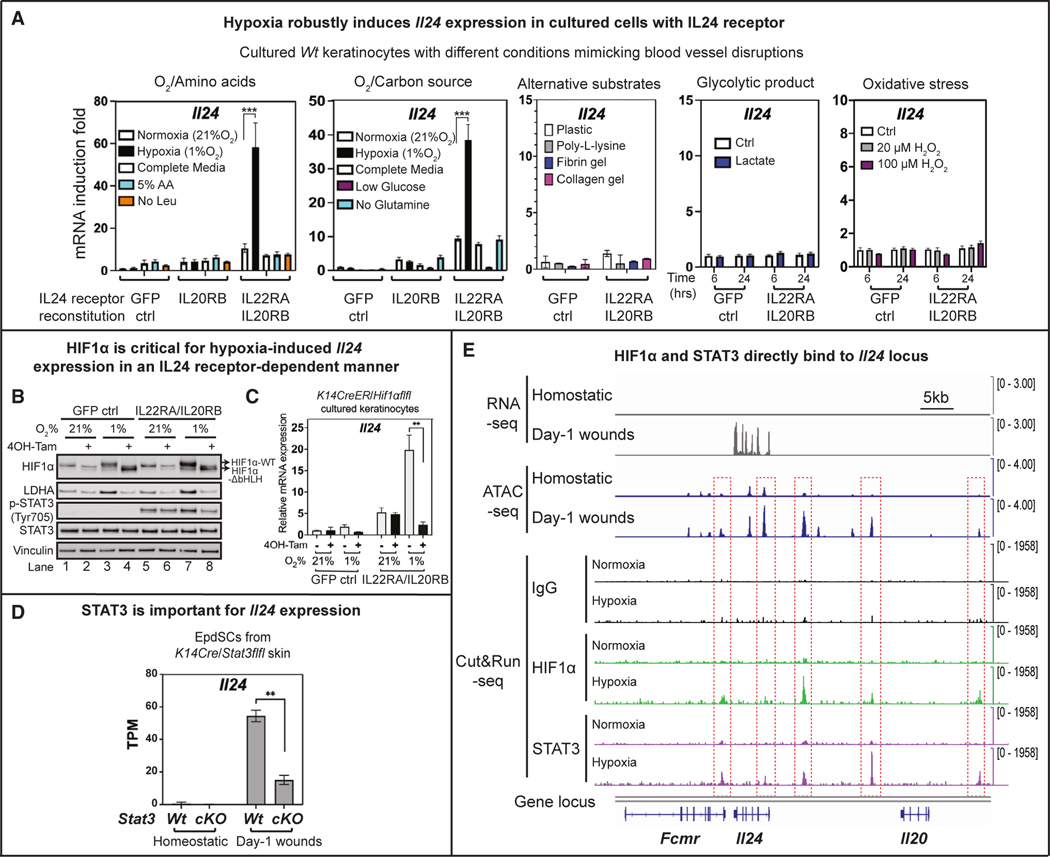
Critical roles for both hypoxia/HIF1α and STAT3 in governing robust *Il24* expression (A) qRT-PCR of *Il24* mRNA in keratinocytes with GFP or IL-24-receptor reconstitution cultured under different oxygen, nutrient, substrate, glycolytic product, and oxidative stress conditions for 48 h. AA, amino acid; Leu, leucine. Note that the native IL-24-receptor, robustly expressed by EpdSCs in their native niche *in vivo*, is silenced under the culture conditions *in vitro*. (B) EpdSCs were isolated from skins of *Krt14CreER*; ${\text{Hif1α}}^{\text{fl/fl}}$ mice, reconstituted with either GFP or IL-24-receptor, and cultured in normoxic (21% O_2_) or hypoxic (1% O_2_) conditions. 4OH-Tam was used to replace the endogenous HIF1α with HIF1α lacking the bHLH DNA binding domain (Hif1α*Δ*exon2). Cells were then immunoblotted for HIF1α, LDHA (lactate dehydrogenase A; encoded by a classical hypoxia-sensitive gene), p-STAT3, STAT3, and vinculin as the loading control. (C) qRT-PCR of *Il24* mRNA in the cells described in (B). (D) *Il24* expression from RNA-seq data performed on FACS-purified EpdSCs from homeostatic skin and day-1 wounds from WT and *Krt14Cre; Stat3*^*fl/fl*^ (*Stat3* cKO)mice treated with 4OH-Tam. TPM, transcripts per kilobase million (n = 3 mice for each genotype). (E) Normalized peaks of RNA-seq, assay for transposase-accessible chromatin sequencing (ATAC-seq), and Cut&Run-seq (with IgG control or antibodies against at HIF1α or STAT3) at the *Il24* locus. Red boxes indicate the 5 chromatin regions at the *Il24* locus that opened upon wounding (ATAC) and have both HIF1α and STAT3 binding peaks (Cut&Run). Peaks from the same experiments are indicated on the same scale. Data in (A), (C), and (D) are presented as mean ± SEM. Sequencing experiments were in duplicates; others were performed ≥3×. Statistical significance was determined using two-tailed unpaired Student’s t tests; *** p < 0.001; and ** p < 0.01. See also Figure S7.

We next explored whether additional possible non-homeostatic patterns associated with blood vessel disruption could induce IL-24. To this end, we established an *in vitro* primary EpdSC culture system and tested a panel of conditions pertinent to blood vessel disruption, including not only hypoxia but also nutrient deprivation (e.g., essential amino acids, glucose, and glutamine), alternative ECM (fibrin clots, collagen), and lactate, a major product of anaerobic glycolysis (Figure 6A). We also tested H_2_O_2_, as it induces oxidative stress, a first signal induced by the wound for immune cell recruitment.^17^

Unexpectedly, none of these *in vitro* conditions, including hypoxia, had a robust effect on *Il24* induction (Figure 6A). This was not because of a culture-related impairment in hypoxia-stabilized HIF1α, as traditional HIF1α targets, *Pgk1* and *Pdk1*,^57^ were induced (Figure S6E). Rather, these results suggested that *Il24* induction after injury requires not only hypoxia and HIF1α but also some additional factor(s). Digging deeper, we learned that despite high expression *in vivo*, IL-24 receptors were silenced *in vitro* (Figure S6F). Upon reconstitution, IL-24-receptor positive keratinocytes responded to hypoxia, but not to the other conditions, in eliciting *Il24* transcription (Figure 6A). Intriguingly, activating *Il24* relied upon not only HIF1α but also IL-24-receptor signaling (Figures 6B, 6C, and S7A).

The downregulation of IL-24-receptor signaling *in vitro* provided a likely explanation for why studies based largely on *in vitro* data have dispensed with IL-24 as either unimportant or counterproductive for epidermal hyperproliferation and wound repair.^21,24^ The existence of a positive receptor signaling feedback loop for *Il24* was reminiscent of that seen for *Ifn*,^36^ and shed light on why following tissue damage, only EpdSCs showed robust *Il24* induction even though many skin cells experienced acute hypoxia and also stabilized HIF1α (Figures 5A and 5B).

Because STAT3 was downstream of IL-24-receptor signaling, we posited that STAT3 might function in concert with HIF1α to regulate *Il24*. Indeed, when we conditionally targeted epidermal *Stat3* and subjected mice to wounding,^13^ *Il24* induction at the wound edge was markedly diminished (Figure 6D). These findings underscored the importance of STAT3 as a major effector of *Il24* in tissue injury and placed IL-24 both upstream and downstream of STAT3. In this regard, *Il24* also differed from classical HIF1α targets, e.g., *Vegfa* and *Ldha* (encoding lactate dehydrogenase A), which showed hypoxia sensitivity and functional HIF1α dependency, but did not rely upon STAT3 for their induction (Figures 6B, S7A, and S7B).

Further addressing the importance for hypoxia/HIF1α on *Il24* expression specifically, we interrogated the effects of IL-17A produced by wound-activated RORC^+^ lymphocytes and recently reported to promote HIF1α stabilization after prolonged hypoxia later in the repair process.^11^ Adaptive immune cells were dispensable for *Il24* induction *in vivo* especially early in the repair process (Figure 2C), and *Rag2/Il2rg* null mice lack IL-17A-producing cells (Figure S7C). That said, under hypoxic conditions *in vitro*, IL-17A boosted *Il24* expression (Figure S7D), revealing an additive, albeit not essential, effect of IL-17A and further underscoring the importance of hypoxia in regulating *Il24*.

Probing deeper, we next examined the wound-induced dynamics of transcription and chromatin accessibility^58^ at the *Il24* locus. Several ATAC (Assay for Transposase-Accessible Chromatin using sequencing)-peaks associated with HIF1α and STAT3 motifs were induced concomitantly with *Il24* transcription at wound-edge EpdSCs (Figures 6E and S7E). Cut&Run sequencing^59^ showed that HIF1α and STAT3 each bound at their cognate sites and in a hypoxia and IL-24 receptor-dependent manner (Figure 6E). In contrast, only HIF1α bound to the *Pgk1* locus, and this canonical hypoxia-induced gene was largely refractile to the status of STAT3 (Figures S7E and S7F). We posit that the dual dependency of *Il24* on both hypoxia and IL-24-receptor signaling ensures specificity and affords fine-tuning in response to tissue damage.

### Additional insights into the role of IL-24 in orchestrating wound repair

**Figure 7.**
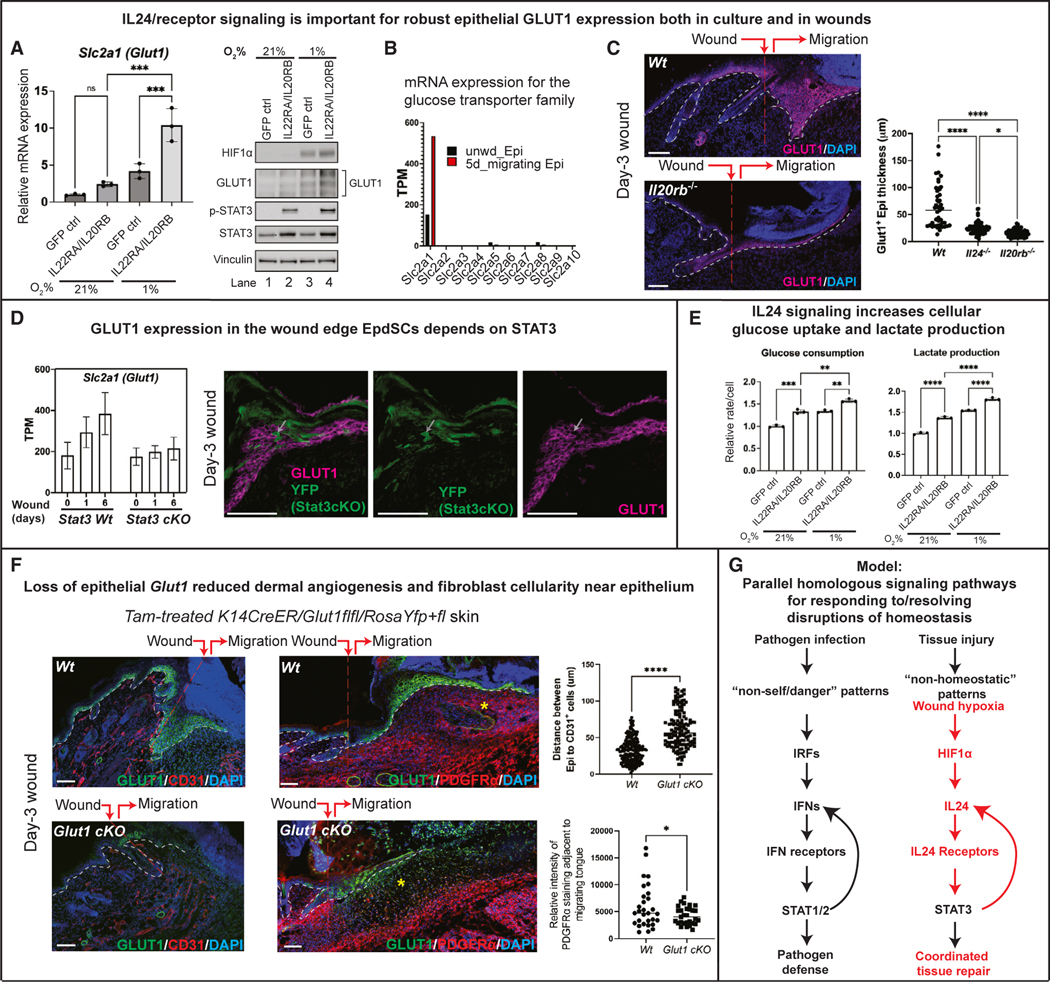
IL-24 signaling promotes epithelial glucose uptake and influences dermal repair (A) GLUT1 expression is dependent upon both hypoxia and IL-24-receptor-signaling. qRT-PCR and immunoblot analyses showing that both events are essential for optimal GLUT1 expression. (B) Glucose transport family expression from RNA-seq performed on EpdSCs that were FACS-purified from homeostatic skin (unwd_Epi) and day-5 wound (5d_migrating Epi). TPM, transcripts per kilobase million. (C) Sagittal sections of day-3 wounds from WT vs. *Il20rb* null skins immunolabeled for GLUT1. Graphs show quantifications of the thickness of GLUT1-expressing epidermis (n = 3 mice per genotype). (D) *Glut1* expression depends upon STAT3. Left: *Slc2a1* mRNA TPM value from RNA-seq of FACS-purified EpdSCs from homeostatic and wounded skin in WT vs. *Krt14-Cre; Stat3flfl* (*Stat3* cKO) mice. Right: sagittal sections of day-3 wounds from WT vs. *Krt14-Cre; Stat3flfl;Yfp+/fl* (*Stat3* cKO) immunolabeled with GLUT1 and YFP (n = 3 mice per genotype). (E) Graphs show relative rates of glucose consumption (left) and lactate production (right) by keratinocytes with GFP or IL-24-receptor reconstitution under normoxic vs. hypoxic conditions. Note that under conditions of hypoxia and IL-24-receptor reconstitution, both measurements are the most elevated. (F) Sagittal sections of day-3 wounds from WT vs. *Krt14Cre; Glut1*^*fl/fl*^ mice treated with topical 4OH-Tam. Sections were immunolabeled for GLUT1 and CD31 (left), or for GLUT1 and PDGFRα (right). Asterisk (*) in the right images denotes a paucity of fibroblasts (${\text{PDGFRα}}^{+}$) in the dermis of *Glut1 cKO* skin. Quantifications at right (n = 6 mice per genotype). (G) Model depicting the similarities between evolutionarily conserved pathogen-induced IFN signaling for defense and injury-induced IL-24 signaling for repair. In contrast to pathogens, which lead to induction of IFN and p-STAT1/2, tissue damage causes hypoxia, leading to HIF1α, IL-24, and p-STAT3. Specifically, EpdSCssense wound hypoxia caused by severed blood vessels, and induce IL-24 and receptor signaling, which subsequently activates STAT3 and further fuels *Il24* expression to promote a coordinated dermal repair and re-epithelialization. The autocrine and paracrine mechanisms underlying wound-induced IL-24-signaling in tissue repair are parallel and functionally analogous to pathogen-induced IFN signaling in pathogen defense, and the two pathways share multiple levels of homology. White dotted lines, epidermal-dermal border; wound site, red dotted line; epidermal migration direction, red arrow. DAPI, nuclei; scale bars, 100 μm. Data in and (C)–(F) are presented as mean ± SEM. Experiments were performed ≥3×. Statistical significance was determined using two-way ANOVA and Tukey’s multiple comparisons tests in (A) and (E), using one-way ANOVA and Tukey’s multiple comparisons tests in figure (C), and using two-tailed unpaired Student’s t tests in (F); **** p < 0.0001; *** p < 0.001; ** p < 0.01; * p < 0.05; and ns, not significant.

Finally, we returned to how the HIF1α-IL-24-STAT3 axis orchestrates the collective involvement of different cells in repairing damaged tissue, this time focusing on downstream transcriptional targets of the axis and their impact on tissue repair. Upon analyzing known hypoxia-induced HIF1α targets for their sensitivity to IL-24-receptor-dependent expression, *Slc2a1*, encoding glucose transporter protein type 1 (GLUT1), stood out (Figures 7A, S6E, and S7B). Moreover, of the glucose transporter family of genes, only *Slc2a1* was expressed strongly in migrating EpdSCs at the wound edge (Figure 7B).

If GLUT1 expression is dependent upon IL-24, then it should show sensitivity to IL-24-receptor activity *in vivo* as well as *in vitro*. Indeed, in both wounded *Il20rb-*null and *Il24-*deficient mice, GLUT1 was diminished (Figure 7C). Moreover, *Glut1* was sensitive to STAT3, as its expression was abolished in *Stat3-*null epidermal cells at the wound edge (Figure 7D).

GLUT1 regulates glucose uptake, leading to elevated lactate production and secretion. We corroborated this effect in our cultured EpdSCs, where the most potent effects on glucose uptake and lactate production were seen under hypoxic conditions and when IL-24-receptor was present (Figure 7E). In contrast to IL-24, lactate can have paracrine effects that don’t require IL-24-receptor signaling, which could explain why macrophages showed positional defects upon IL-24 loss, even though they appeared to lack IL-24-receptor/p-STAT3-signaling. Lactate can also have a proangiogenic effect on macrophages,^60,61^ raising the possibility of additional signaling circuits unleashed downstream of IL-24-receptor-signaling. That said, conditional ablation of *Glut1* in EpdSCs *in vivo* had on its own a hitherto unappreciated impact on both paracrine effect on angiogenesis and fibroblasts close to the epidermis at the wound edge (Figure 7F).

These evidences, combined with our observation that IL-24 directly signals to dermal endothelial cells and fibroblasts (Figure S3G), suggest that by inducing IL-24 in response to injury, EpdSCs orchestrate both autocrine and paracrine cascades of events involving proliferation and metabolic changes that together trigger a joint collaboration among tissue cells to orchestrate coordinated repair after injury.

## DISCUSSION

Injury and infection are universal insults to living organisms throughout evolution. The ability to properly sense and respond to acute insults for timely resolution is essential for organismal survival. Numerous PAMPs are known to stimulate IFN signaling to resist infection.^2^ Here, we uncovered a previously elusive molecular pathway that is induced upon tissue damage, independent of microbes and the adaptive immune system (Figure 7G). At the root of this tissue damage pathway is an IFN homolog, IL-24, which while not expressed in homeostasis, is specifically induced by EpdSCs at the hypoxic wound edge region. The ability to sense tissue damage such as hypoxia in a microbe- independent manner distinguishes IL-24 from PAMP-induced signaling. However, analogous to the role of IFN in resisting pathogen infection, IL-24 coordinates a pro-angiogenetic repair and proliferation program to restore tissue integrity and homeostasis.

IFN production must be tightly regulated to prevent inflammation and autoimmunity.^36,62^ We learned that IL-24 production is similarly tightly regulated and occurs only at the wound site. Although the damaged blood vessels generate a hypoxic state, hypoxia alone was not sufficient for *Il24* activation, which also relied upon autocrine IL-24-receptor signaling and STAT3 activation. The feedback loop that we exposed here provides an interesting insight into how the epithelial tongue progresses specifically at the wound site and how it is able to simultaneously coordinate dermal repair in proximity. In the end, the repair process becomes naturally autoregulated at the back end in that as the vasculature is re-established, both the hypoxia-induced signaling and *Il24* expression wane.

Our data revealed that as an epithelial-derived cytokine induced at the wound site, IL-24 is poised to unleash a multifaceted cascade of paracrine and autocrine effects in coordinating tissue repair. Although IL-24-receptor expression is highest in EpdSCs, nearby dermal endothelial cells and fibroblasts also express the receptor and directly proliferate in response to IL-24. Additionally, however, IL-24 also alters gene expression through its ability to activate STAT3 signaling, and downstream effectors such as the glucose transporter GLUT1. Although GLUT1 has been shown to impact epidermal proliferation and wound re-epithelialization,^11,63^ we discovered that GLUT1 is highly upregulated in the wound edge epithelium, where it is impacted directly by autocrine IL-24-receptor signaling. IL-24’s ability to alter epithelial metabolic processes, including lactate production to impact mesenchymal repair response within the wound bed takes on newfound importance, as it suggests that IL-24’s paracrine effects may extend beyond whether a cell within the injured skin expresses the IL-24-receptor.

In closing, the mechanistic insights we have unraveled here strongly suggest that by sensing injury signals such as hypoxia and autocrine IL-24-receptor/STAT3 signaling to maximize IL-24 production, EpdSCs not only choreograph their own proliferation and re-epithelialization to seal wounds but also coordinate the requisite dermal repair responses that involve blood vessel sprouting and fibroblast reconstruction of the ECM. Our findings also offer insights into complex infectious and inflammatory diseases, which can cause secondary tissue damage, the proper repair of which is essential for disease tolerance and host survival.^64,65^ In this regard, it is intriguing that in severe COVID-19 cases, patients with damaged lungs display prominent IL-24,^66^ and the colons from patients with ulcerative colitis also express IL-24.^49^ Taken together, the implications of our findings here are likely to extend broadly to many conditions of tissue damage.

### Limitations of the study

Further investigations will be needed to fully dissect the myriad of possible secondary effects that are likely to be triggered downstream of IL-24 signaling. Given the lack of *Il20rb*-floxed mice and the complexity of cell types involved, a comprehensive study of IL-24 signaling in each cell type within the wound bed was beyond the scope of the current study. Methodology is currently limited for measuring the *in vivo* levels of lactate and other metabolites in homeostasis and at wound sites. We mostly limited our studies to female animals, as males tend to fight and introduce wounds that might preclude accurate analyses.

## STAR★METHODS

### RESOURCE AVAILABILITY

#### Lead contact

Further information and requests for resources and reagents should be directed to and will be fulfilled by the lead contact, Elaine Fuchs (fuchslb@rockefeller.edu).

#### Materials availability

Materials used in this study will be provided upon request and available upon publication.

#### Data and code availability

- Bulk RNA-, 10x singe-cell RNA-, ATAC-sequencing data and Cut-and-Run sequencing data from this study have been deposited in the Gene Expression Omnibus (https://www.ncbi.nlm.nih.gov/sra) under accession codes PRJNA731164, PRJNA885018, and PRJNA731304. All other data in the manuscript, supplementary materials and source data are available from the corresponding author upon request.
- All original code is available from the lead contact upon request.
- Any additional information required to reanalyze the data reported in this paper is available from the lead contact upon request.

### EXPERIMENTAL MODEL AND SUBJECT DETAILS

#### Animals

C57BL/6 and *B6.129X1-Gt(ROSA)26Sor^tm1(EYFP)Cos^/J* (*Rosa26-stop-lox-stop YFP*) mice were purchased from The Jackson Laboratory. *Krt14-Cre* and *Krt14-CreER* mice were previously generated in the Fuchs laboratory. Il20rb^−/−^ mice were obtained from Genentech, which was previously used in a skin wound healing study.^82^ Hif1α null mice were obtained by crossing Hif1α floxed animals from The Jackson Laboratory (Stock No: 007561) to *Krt14-CreER*/*Rosa26-YFP* (Fuchs Lab) animals. *Glut1* null mice were obtained by crossing *Glut1* floxed animals from The Jackson Laboratory (Stock No: 031871) to *Krt14-CreER/Rosa26-YFP* (Fuchs Lab) animals. *Stat3* cKO mice were obtained by crossing Stat3 floxed animals from The Jackson Laboratory (Stock No:016923) to *K14-Cre/ Rosa26-YFP* (Fuchs Lab) animals. *Myd88^−/−^*(Stock No: 009088) and *Trif^−/−^* (Stock No: 005037) mice were obtained from Jackson Laboratories and crossed into *Myd88^−/−^Trif^−/−^* in-house. *Rag2^−/−^Il2rg^−/−^* (Stock No. 4111-F) and control wildtype C57BL/6NTac (Stock No. B6-F) females were purchased from Taconic. TNFR1/TNFR2 DKO mice were purchased from The Jackson Laboratory (Stock No. 003243).

In order to generate *Il24^−/−^* mice using the CRISPR-Cas9 method, we used the Alt-R CRISPR-Cas9 system from IDTdna. *Il24* gRNA (GGAGAACCACCCCTGTCACT) targeting its exon 2 was selected using guidescan (http://www.guidescan.com/). crRNA (containing *Il24* gRNA sequence), tracrRNA (IDT cat. #1072533), and recombinant Cas9 (IDT cat. #1081058) were purchased from IDTdna, and crRNA:trRNA:Cas9 RNP particles were assembled *in vitro* as described by the manufacturer and suspended in injection buffer (1 mM Tris-HCl pH 7.5, 0.1 mM EDTA) at a final RNP concentration of 0.122 μM. The mixture was then injected into the pronucleus of fertil- ized single-cell mouse embryos, and embryos were implanted into the oviducts of pseudo-pregnant wild-type C57BL/6 female mice.^83^ For the generation of mice with inducible *Il24* loss of function specifically in skin epithelium, we used the sleeping beauty sys- tem and mir-E based shRNA method.^84^ For TRE-inducible *Il24* knockdown *in vivo*, we designed *Il24* shRNA with the algorithm from splashRNA,^85^ and cloned the shRNA with the optimal antisense sequences (TAGAATTTCTGCATCCAGGTCA) into the mir-E backbone^86^ placed at the 3’UTR of a nucleus-localized H2B-GFP reporter driven by a TRE promoter. After validation of efficient knockdown in keratinocytes *in vitro*, the TRE-H2B-GFP-shIl24 cassette was cloned into a sleeping beauty transposon (Addgene Plasmid #108352) for injection into the zygotes of *K14rtTA* mice.^87^ The transposon plasmid was then mixed with a plasmid encoding trans- posase (pCMV-SB100; Addgene Plasmid #34879) in injection buffer (2.5 ng/μl transposon plasmid; 1.25 ng/μl SB100 transposase plasmid; 5 mM Tris-cl pH 7.4, and 0.1mM EDTA), and injected into the pronucleus of fertilized single-cell mouse embryos of *K14rtTA*, and embryos were implanted into the oviducts of pseudo-pregnant C57BL/6 female mice. Once the sleeping beauty mice were born, female mice and control littermates were subjected to wounding experiments, while male mice with high transduc- tion efficiency were used as founder mice to back-cross with *Krt14-rtTA* C57BL/6 female mice to generate F1 offspring mice.

Animals were assigned randomly to experimental groups and studies were not blinded. However, age- and sex-matched, and whenever possible, littermates were used for each experiment. For the full-thickness wound healing time course and wound imaging experiments, female mice in the telogen phase of the hair cycle (P50-P65) were used, as males tend to fight and introduce wounds that might preclude accurate analyses. Mice were maintained in the Association for Assessment and Accreditation of Laboratory Animal Care-accredited animal facility of The Rockefeller University (RU), and procedures were performed with Institutional Animal Care and Use Committee (IACUC)-approved protocols. Mice of all strains were housed in an environment with controlled temperature and humidity under specific-pathogen-free conditions, on 12 hour-light:dark cycles, and fed with regular rodent’s chow or doxycycline as described.

#### Cell lines

293TN HEK cells for lentiviral production were cultured in DMEM medium with 10% FCS (Gibco) and 1 mM sodium pyruvate, 2 mM glutamine, 100 units/mL streptomycin, and 100 mg/mL penicillin.

#### Primary cell cultures

Primary epidermal stem cells (EpdSCs) were maintained at 37°C in a humidified atmosphere containing 7.5% CO_2_. Cells were cultured in E-low calcium (50 μM Ca^2+^) medium made in-house from DMEM/F12 (3:1 ratio) medium supplemented with 15% chelated FBS, 5 μg/mL insulin, 5 μg/mL transferrin, 2 nM triiodothyroxine, 40 μg/mL hydrocortisone, 10 nM cholera toxin and Pen-Strep.^88^ C57BL/6 mouse primary dermal microvascular endothelial cells were purchased from Cellbiologics (C57–6064) and pure CD31^+^ blood endothelial cells were FACS-purified based on markers endomucin, CD31, PDPN and LYVE1.^89^ The purified blood endothelial cells were then cultured in commercially available endothelial media from Cellbiologics (M1168) containing 5% FBS. Primary fibro- blasts were cultured in DMEM:F12 (3:1) containing Pen-Strep and 10% FBS.

### METHOD DETAILS

#### Cell culture experiments

For *in vitro* hypoxia experiments, primary EpdSCs with GFP or IL24-receptor reconstitution were cultured under 21% oxygen (normoxia) or 1% oxygen (hypoxia) in DMEM/F12 (3:1 ratio) medium supplemented with 15% chelated FBS, 5 μg/mL insulin, 5 μg/mL transferrin, 2 nM triiodothyroxine, 40 μg/mL hydrocortisone, 10 nM cholera toxin and Pen-Strep.^88^ For the generation of each nutrient-deprived condition, amino acid/glucose/glutamine deficient DMEM/F12 (complete deficient media) was made in-house by the MSKCC media core (dialyzed chelated FBS was used), and reconstituted with each nutrient, and the complete medium re- supplemented with all missing nutrients served as a control. Cells were also cultured on the plates coated with poly-L-lysine, fibrin, or collagen as indicated, according to the manufacturer’s instructions. For IL17A stimulation, IL24-receptor reconstituted keratinocytes cells were cultured either under 21% oxygen (normoxia) or 1% oxygen (hypoxia) conditions, with 10ng/ml or 100ng/ml recombinant IL17A for 4 days to mimic chronic hypoxic conditions in the later wound edge. For IL24 stimulation, both endothelial cells and fibroblasts were cultured in low serum condition (1%) for 6 hours before stimulation, followed by 100 ng/ml IL24 treatment for 40 minutes. EdU was added to the culture 15 minutes before harvest.

#### Metabolic analysis *in vitro*

For measuring glucose uptake and lactate production, GFP control and IL24-receptor reconstituted keratinocytes were plated in triplicates in 12-well plates at 50,000 cells/well and were allowed to attach overnight in E-low calcium medium. The next day, following same media change, cells were placed in normoxic or hypoxic conditions overnight. Media glucose consumption and lactate production were then measured using the YSI 2900 analyzer and normalized by cell number.

#### IL24-receptor reconstitution and Hif1α KO cells

For IL24-receptor reconstitution, either a GFP control or a mouse cDNA encoding IL20RB was cloned into pTY-EF1A-puroR-2a lentiviral vector, and either a GFP control or a mouse cDNA encoding IL22RA1 were cloned into pTY-EF1A-HygromycinR-2a lentiviral vector. Lentivirus was packaged in 293TN cells and then used to infect wild-type or *Krt14*CreER^+^;Hif1αfl/fl keratinocytes, which were selected by puromycin 1 μg/ml and hygromycin 50 μg/ml for a week. For floxing out Hif1α exon2 in *Krt14*CreER^+^; Hif1αfl/fl cells to generate Hif1α loss of function cells, 3 μM 4-Hydroxytamoxifen (4OH-Tam) was added to the culture for 4 days. Alternatively, guide RNA targeting Hif1α ^90^ was cloned into pLentiCRISPRv2-blasticidin construct (Addgene Plasmid #98293). Lentivirus was packaged in 293TN cells and then used to infect GFP control or IL24-receptor reconstituted keratinocytes, which were selected by blasticidin (3 μg/ml, InvivoGen) for 4 days prior to the experiments.

#### Full-thickness wounding

Punch biopsies were performed on anesthetized mice in the telogen phase of the hair cycle (P50-P65).^91^ For wounding the back skin, dorsal hairs were shaved with clippers and skin was swabbed with ethanol prior to wounding. 4mm or 6 mm biopsy punches (Miltex) were used to make full-thickness wounds. After wounding, tissues were collected at 1, 3, 5 or 7 days after wounding as indicated.

#### Immunofluorescence microscopy

Mouse back skin was dissected, fixed with 4% paraformaldehyde diluted in PBS for 1–2 hours at 4°C, washed with PBS three times, incubated with 30% sucrose at 4°C overnight, and then embedded in OCT (Tissue Tek). Frozen tissue blocks were sectioned at 14 μm on a cryostat (Leica) and mounted on SuperFrost Plus slides (Fisher). The tissue sections were blocked for 1 hour at room temperature with the blocking solution (5% normal donkey serum, 0.5% bovine serum albumin, 2.5% fish gelatin, and 0.3% Triton X-100 in PBS). Sections were then incubated with the indicated primary antibodies diluted in the blocking solution at 4°C overnight. For staining the tissues with an anti-p-STAT3 or an anti-HIF1α antibody, the sections were pretreated with ice-cold 100% methanol prior to the blocking step. The sections were then washed three times with 0.3% Triton X-100 in PBS and incubated with secondary antibodies diluted in the blocking solution at room temperature for 1 hour. Finally, the sections were washed three times with 0.3% Triton X-100 in PBS, three times with PBS containing DAPI at a 1:3,000 dilution, and then mounted with ProLong Dimond Antifade Mountant (Thermo Fisher Scientific). EdU click-it reaction was performed according to the manufacturer’s instructions (Life Technologies) after the secondary antibody incubation and was followed by washing with PBS containing DAPI, as needed. The samples were visualized with an AxioOberver.Z1 epifluorescence microscope equipped with a Hamamatsu ORCA-ER camera and an ApoTome.2 (Carl Zeiss) slider. Tiled and stitched images of sagittal sections were collected using a 20X objective, controlled by Zen software (Carl Zeiss). Alternatively, whole wound images were captured using a BioTek Cytation 5 using a 4x air objective. In order to present a larger wounded area, most of the immunofluorescence images presented (except for Figure 4E, 7D and S3G) were tiled images taken by either AxioOberver.Z1 or Biotek Cytation 5 automatically, and were then stitched into bigger images by respective software Zen (Zeiss) or Gen5 (BioTek). Please note some of the images such as Figure S6B may still show a straight line in between two stitched single images due to imperfect shading correction after processed by Zen. BioTek images did not show such shading correction problem. ImageJ software was used to project Z-stacks and process images. The size of the images was adjusted and assembled in Adobe Illustrator. Scale bars were indicated in the figures and legends. Antibodies against following mouse pro- teins were used for immunofluorescence staining in the study: p-STAT3 (rabbit, Cell Signaling), HIF1α (rabbit, Cell Signaling), GLUT1 (rabbit, Abcam), CD31 (rat, Biolegend), Endomucin (Rat, Santa Cruz), GFP (chicken, Abcam), PDGFRa/CD140a (Rat, Biolegend), Intergrin-α5/CD49e (Rat, Biolegend), Krt14 (Chicken, Biolegend), Collagen-I (Rabbit, Abcam), CD31 (Hamster, Millipore), Ki67(Rabbit, Cell Signaling), ARG1(Goat, Novusbio), MHCII(Rat, Biolegend), VEGFA(Goat, R&D Systems).

#### Whole-mount immunostaining for wounded skin

For adult skin wounds, the entire wound bed and 1 mm of skin surrounding the wound were dissected from the back skin and placed on Whatman paper. The tissue was then soaked in PBS for half an hour, and the scab was gently removed if needed, and excess fat tissue was gently removed from the dermis side using sharp forceps. The wounded tissues were then fixed in 4% PFA in PBS for one hour at room temperature, followed by extensive washing in PBS. Tissues were then permeabilized for at least 5 hours (and up to overnight) in 0.3% Triton X-100 in PBS, followed by blocking buffer (2.5% fish gelatin, 5% normal donkey serum, 3% BSA, 0.3% Triton) for additional 2 hours. For immunolabeling, primary antibodies (Krt14, 1:500; Endomucin, 1:300) were incubated at room temperature for two days, followed by extensive washing with 0.3% Triton X-100 in PBS. Samples were then incubated for additional two days at room temperature with secondary antibodies conjugated with Alexa 488, RRX, or 647 (1:500 Life), and DAPI (0.2 μg/ml; 1:500). Samples were washed with 0.3% Triton X-100 and DAPI (1:500) in PBS for 4 hours at room temperature and proceeded to tissue clearing.

#### Tissue clearing

Tissue clearing was performed as previously described with some modifications.^44^ Stained back skin tissues were transferred through increasing concentrations of ethanol diluted in molecular grade water and adjusted to pH 9.0: 30%, 50%, and 70% for 2 hours each, all at room temperature under gentle shaking. Dehydrated tissues were then incubated for two rounds of 100% ethanol for 2 hours each, at room temperature under gentle shaking, before transferring into 1 ml ethyl cinnamate in Eppendorf tubes (polypropylene) for clearing. Cleared skin was mounted with ethyl cinnamate drops between 2 cover glass sizes 22×40 mm, #0 (Electron Microscopy Science), and placed in the microscope slide holder to acquire images. Images could be acquired within 30 min of tissue clearing or up to 3 months of staining and clearing.

#### Proximity ligation *in situ* hybridization

Proximity ligation in situ hybridization technology (PLISH) is performed as previously described^33^ with slight modifications. Mouse skin samples were fixed with 4% paraformaldehyde in DEPC-treated PBS at 4°C for 1 hour, rinsed three times with DEPC-treated PBS, incubated with DEPC-treated 30% Sucrose/PBS solution for a few hours, and embedded in OCT. 10 μm tissue sections were prepared from frozen OCT blocks, pretreated with 25 μg/ml pepsin in 0.1 M HCl at 37°C for 5 minutes, and rinsed with DEPC-treated PBS. After drying at room temperature for approximately 5 minutes, tissue sections on the microscope slides are sealed with adhesive chambers (Grace Bio-Labs, GBL622514), rinsed with Hybridization Buffer (1 M NaTCA, 5 mM EDTA, 50 mM Tris pH 7.4, 0.2 mg/ mL Heparin, and 0.1% LDS in DEPC-treated water), and incubated with a mixture of hybridization probes (sequences listed below, 100 nM final concentration each) in Hybridization buffer at 37°C. After a 2 hour-incubation in a humid hybridization oven, the tissue sections were rinsed four times with Hybridization Buffer, incubated with High Salt Buffer (0.25 M NaCl, 50 mM Tris, 2 mM EDTA, and 0.1% LDS in DEPC-treated water) at 37°C for 10 minutes, rinsed once with Circle Hybridization Buffer (2x SSC/20% Formamide, 0.2 mg/mL Heparin, and 0.1% LDS in DEPC treated water), and incubated with 116 nM phosphorylated Common Connector Circle (CCC) oligo and phosphorylated Variable Bridge (VB) oligo (sequences listed below) in Circle Hybridization Buffer at 37°C in a humid hybridization oven. After a 1 hour-incubation, the tissue sections were rinsed twice in Circle Hybridization Buffer, once with 1x T4 DNA ligase buffer (NEB, B0202S) in nuclease-free water (Invitrogen, AM9937), and incubated with a ligation reaction mixture (10 unit/μL T4 DNA ligase (NEB, M0202M), 1x T4 DNA ligase buffer, 0.4 μg/μL BSA, 0.4 unit/μL RNaseOUT (Invitrogen, 10-777-019), 250 mM NaCl, 0.005% Tween-20 in nuclease-free water) at 37°C in a humid hybridization oven. After a 2 hour-incubation, tissue sections were rinsed twice with Circle Hybridization Buffer, rinsed once with 1x phi29 polymerase buffer (Lucigen, NxGen kit 30221) in nuclease-free water, and incubated with a rolling-circle amplification (RCA) reaction mixture (1 unit/μL phi29 polymerase (Lucigen, NxGen kit 30221), 1x phi29 polymerase buffer, 5% Glycerol, 0.25 mM each dNTP, 0.4 μg/μL BSA, 0.4 unit/μL RNaseOUT in nuclease- free water) at 37°C in a humid hybridization oven. After overnight (~16 hours) RCA reaction, the tissue sections were rinsed twice with Label Probe Hybridization Buffer (2x SSC/20% Formamide, 0.2 mg/mL Heparin in nuclease-free water) and incubated with 50 nM Label Probe (sequence listed below) in Label Probe Hybridization Buffer at 37°C in a humid hybridization oven for 2 hours. The labeled samples were washed twice with 0.05% Tween-20 in DEPC-treated PBS, stained with 1 μg/ml DAPI in DEPC-treated PBS, rinsed with DEPC-treated PBS, and imaged on the MIDAS microscope. The DNA oligos used for PLISH were purchased from Eurofins Genomics. The sequences (from 5’ to 3’ end) are listed below:

-CCC (5’ phosphorylated, HPLC purification): ATTCCTGACCTAACAAACATGCGTCTATAGTGGAGCCACATAATTAAACCTGGCTAT

-VB (5’ phosphorylated, HPLC purification): ACTACTCGACCTATAACCATAACGACGTAAGT

-Label Probe (5’ conjugated with Alexa Fluor 647, HPLC purification): ACTATACTACTCGACCTATA

-Design of H probes:

Il24-H1L: AGGTCAGGAATACTTACGTCGTTATGGAGGGTCCTAAAGTGAAGCCG

Il24-H1R: AAAGGGCCAGTGCTCCTGCTTTATAGGTCGAGTAGTATAGCCAGGTT

Il24-H2L: AGGTCAGGAATACTTACGTCGTTATGGAGGCTCAGGCAGGGGAGAAT

Il24-H2R: GGTTCCAAAGAAGAAGGATTTTATAGGTCGAGTAGTATAGCCAGGTT

Il24-H3L: AGGTCAGGAATACTTACGTCGTTATGGTCACTAATGGGAAGCATGGA

Il24-H3R: AAAACCGCTGGTGTGCACTCTTATAGGTCGAGTAGTATAGCCAGGTT

Krt14-H1L: AGGTCAGGAATACTTACGTCGTTATGGTGGCGGTTGGTGGAGGTCAC

Krt14-H1R: CCATGACCTTGGTGCGGATCTTATAGGTCGAGTAGTATAGCCAGGTT

Krt14-H2L: AGGTCAGGAATACTTACGTCGTTATGGAAAGAGTGAAGCCTATAGGG

Krt14-H2R: AGGAAGGACAAGGGTCAAGTTTATAGGTCGAGTAGTATAGCCAGGTT

#### Evolutionary analysis of cytokines/receptors

We retrieved the protein family containing IL24 from Pfam and ECOD databases.^92,93^ Pfam classifies proteins using sequences while ECOD takes similarity in protein structure into consideration. IL24 belongs to the Pfam family IL10 (PF00726), which is a member of the Pfam clan 4H cytokine (CL0053). 4H cytokine clan is equivalent to the 4-helical cytokine homologous group of ECOD, and we included all the 29 Pfam families from this clan in our study. We identified Pfam domains in each human protein from Uniprot using HMMER (e-value < 0.00001).^94,95^ A total of 59 human proteins contained Pfam domains from the 4H cytokine clan, and we extracted the sequences of these domains and aligned them using PROMALS3D^96^ (Table S3). The multiple sequence alignment (MSA) of these Pfam domains were used for phylogenetic analysis by RAxML (-m PROTGAMMAAUTO).^97^ After initial alignment, we picked representative cytokine from each clade that highlighted in yellow from Table S3, and used the same method to generate a smaller phylogenic tree for presentation.

We identified the receptors for all human cytokines based on literature (Table S3). We identified Pfam domains in these cytokine receptors using HMMER and found that majority (35 out of 40) of them contain >=2 tandem immunoglobulin-like (Ig-like) domains in their extracellular regions. We built MSA for two Ig-like domains from these receptors using the following approach. First, we focused on receptors containing two Ig-like domains and obtained the MSA of the tandem Ig-like domains in these receptors. Second, for each cytokine receptor with >= 3 Ig-like domains, we iterated all combinations of two Ig-like domains from it and identified the combination showing maximal sequence similarity, measured by BLOSUM55 matrix to the MSA we built in the first stage. We extracted regions for the best combination for each receptor and concatenated the sequences for the two Ig-like domains to represent this receptor. Finally, we aligned the sequences of two representative Ig-like domains from all the receptors with >= 2 such domains using PROMALS3D, and the resulting MSA was used to reconstruct the phylogeny of these receptors through RAxML.

#### Germ-free mice wounding

Germ-free (GF) C57BL/6 wild-type (WT) mice were kept in germ-free flexible film isolators (Class Biologically Clean Ltd) at Rockefeller University. For wounding experiments, GF C57BL/6 mice were exported to isocages bioexclusion system (Tecniplast, PA, USA) and housed in isocages for the duration of the experiment. Wounding of GF mice was performed in a sterile hood using sterile autoclaved instruments. Wounding of specific-pathogen-free (SPF) C57BL/6 WT mice was performed in the same hood after GF mice were transferred into the isocages. Both GF and SPF mice were then housed in the isocages under the same conditions for 1 or 5 days as described before harvesting skin wounds. Mice housed in the isocages were provided with autoclaved food and water.

#### Flow Cytometry Analysis and Cell Sorting

In order to isolate and stain EpdSCs from the homeostatic mouse back skin, subcutaneous fat was removed from the skin with a scalpel, and the skin was placed dermis side down on 0.25% trypsin (Gibco) and 0.1 mg/ml DNase at 37 °C for 45 minutes while shaking gently. For isolating Day-1 wound edge EpdSCs, skin wounds were first excised at about 1–2 mm from the wound edge. Subcutaneous fat was then removed, and the skin was placed on a Whatman filter paper, faced down to be soaked entirely in trypsin, and incubated for 15–18 minutes while shaking gently. For Day-5 or 7 wounds, wounds were excised at 1 mm from the wound edge, placed on a Whatman filter paper, faced down to be soaked entirely in 50 mM EDTA in PBS, and incubated at 37°C for 1 hour while shaking gently. After the incubation, the scabs were firstly removed, the wound edge epidermis including the migrating tongue was then carefully dissected and isolated from the dermis under a dissection microscope. The isolated epidermis was then incubated in trypsin for about 12 minutes while shaking gently. Single-cell suspensions were obtained by scraping the skin to remove the epidermis and hair follicles from the dermis of homeostatic skin or Day-1 wounds. Single-cell suspensions for Day-5 or 6 wounds were obtained by pipetting the suspension to release single cells. Cell suspensions were then filtered through 70 mm, followed by 40 mm strainers. Cell suspensions were incubated with the indicated antibodies for 30 minutes on ice. The following anti-mouse an- tibodies were used for FACS: α6-integrin-PE or BV650 (BD Pharmingen, 1:1,000), CD34-efluor660 or BV421 (eBiosciences, 1:100), Sca-1-PerCP-Cy5.5 (Biolegend, 1:1,000), CD45-APC-Cy7 (Biolegend,1:200), CD31-PE-Cy7 (Biolegend,1:300), biotin-CD117 (Bio- legend, 1:200), CD140a-APC (Biolegend, 1:100), Streptavidin- PE-Cy7 (eBioscience, 1:500), CD90-BV421 (Biolegend, 1:200). For biotin-conjugated primary antibodies, after washing with FACS buffer, cells were incubated with Streptavidin PE-Cy7 (1:500). DAPI was used to exclude dead cells. Cell isolations were performed on BD FACSAriaII SORP running BD FACSDiva software (BD Biosciences). Flow Cytometry Analyses (data acquisitions) were performed using BD LSRFortessa and BD LSRII analyzers running BD FACSDiva software, and the data were analyzed with FlowJo software (BD Biosciences).

For the analysis of dermal cells at the wound site, wound tissue was isolated from the back skin, keeping margins as close as 1 mm. The whole wounds were first excised and placed on a Whatman filter paper, faced down to be entirely soaked in PBS for half an hour, softened scabs were then carefully removed to expose live tissue underneath. Tissue was minced in media (RPMI with L-glutamine, β-mercaptoethanol, sodium pyruvate, acid-free HEPES, penicillin and streptomycin), added with Liberase TL (Roche; 250 μg/ml) and 0.1 mg/ml DNase, and digested for 60–90 minutes at 37°C while shaking gently. The digest reaction was stopped by adding 20 μl of 0.5 M EDTA. Single-cell suspensions were then obtained by pipetting the suspension to release single cells. Cells were filtered through a 70 μm strainer, and then a 40 μm strainer. For 10x single cell RNA-seq, the cell suspensions were additionally incubated with ACK lysing buffer (Thermofisher) to remove red blood cells, and then live, single cells were sorted after adding DAPI.

Cell suspensions for other analysis and sorting experiments were then stained with the following antibodies from Biolegend: α6-integrin-PE (1:1,000), CD45-APC-Cy7 (1:200), CD31- PE-Cy7 (1:300), CD11b-BV421 (1:1,500), MHCII-AF700 (1:1,000), CD45-APC-Cy7 (1:200), CD140a-APC (1:100), ITGA5-Ax488 or APC (1:100), Ly6G-PE or APC (1:500). In particular, for the wound bed innate immune cell panel analysis, we used the following combination: CD45-APC-Cy7 (Biolegend, 1:200), CD117-PerCP-Cy5.5 (Biolegend 1:200), Ly6C-FITC, (Biolegend, 1:200), Ly6G-PE (Biolegend, 1:200), Siglec F-APC (Biolegend 1:200), FceRIa-PE-Cy7 (eBioscience, 1:200), CD64-BV605 (Biolegend 1:200), CD11b-BV421 (Biolegend 1:200), MHCII-(I-A/I-E) AF700, (Biolegend 1:200). Dead cells were excluded using a LIVE/DEAD Fixable Blue Dead Cell Stain Kit (Molecular Probes) or DAPI. Flow Cytometry Analyses (data acquisitions) were performed using BD LSRFortessa and BD LSRII analyzers running BD FACSDiva software, and the data were analyzed with FlowJo software (BD Biosciences).

#### Bulk RNA-seq and quantitative RT-PCR

Total RNA from sorted EpdSCs, endothelial cells, dermal fibroblasts, and innate immune cells was purified using the Direct-zol RNA MiniPrep kit (Zymo Research) per the manufacturer’s instructions. DNase treatment was performed to remove genomic DNA (RNase-Free DNase Set, Qiagen). The quality of RNA samples was determined using an Agilent 2100 Bioanalyzer, and all samples for sequencing had RNA integrity (RIN) numbers >8. cDNA library construction using the Illumina TrueSeq mRNA sample preparation kit was performed by the Weill Cornell Medical College Genomic Core facility (New York, NY), and cDNA libraries were sequenced on an Illumina HiSeq 2000 or Illumina Novaseq 6000 instruments.

The bulk RNA-seq data analysis was mainly processed in R (version 4.0) environment. The reference genome sequence was fetched from BSGenome.Mmusculus.UCSC.mm10 package (https://bioconductor.org/packages/release/data/annotation/html/BSgenome.Mmusculus.UCSC.mm10.html ); the GTF file was fetched from TxDb.Mmusculus.UCSC.mm10.knownGene package (https://bioconductor.org/packages/release/data/annotation/html/TxDb.Mmusculus.UCSC.mm10.knownGene.html). The fastq files were aligned to reference genome by Salmon (version 1.4.0, https://salmon.readthedocs.io/en/latest/salmon.html), and the counts for each feature were calculated by Salmon. The counting results were imported into DESeq2 object by tximport (https://bioconductor.org/packages/release/bioc/html/tximport.html ). For real-time PCR, equivalent amounts of RNA from FACS-purified cells were reverse-transcribed using the SuperScript^™^ VILO^™^ cDNA Synthesis Kit (ThermoFisher Scientific). All cDNAs were normal- ized to equal amounts using housekeeping genes *Eef1a1* and *Ppib*. If not specified in the figure legends, data normalized to *Eef1a1* are presented and similar expression trends were also confirmed with *Ppib*. cDNAs were mixed with indicated gene-specific primers and SYBR green PCR Master Mix (Sigma), and qRT-PCR was performed on an Applied Biosystems 7900HT Fast Real-Time PCR system.

#### 10x single-cell RNA-seq analysis

The raw fastq files of 10X data were mapped to mouse genome (mm10), and the gene expression of each gene in each cell was estimated by the count function of Cell Ranger (v 3.0.2). The counting matrices of the two samples were then merged by the aggr function of Cell Ranger. The “.cloupe” file was applied for data visualization with Loupe Browser (v 3.0.0).

More customized analyses were processed by Seurat (v 3.0.0) which was developed on R language (version 3.5.2). The following steps were derived from Seurat vignette. First, the filtered counting matrices of the samples were loaded into Seurat object. The features detected in less than five cells were removed. The proportion of mitochondrial genes oriented UMI counts (percent.mt) was also estimated. Then, the Seurat object was subjected to log normalization (Seurat::NormalizeData) and variable features identification (Seurat::FindVariableFeatures). After this step, amount 2000 variable features were identified by vst method. To merge the Seurat objects for all samples, the CCA-based workflow was applied. After merging all samples, the cells with the following criteria were removed: (i) too few genes detected (nFeature_RNA < 200); (ii) potential doublets (nCount_RNA > 99% quantile of UMI counts); potential cell debris (percent.mt > 10%). After removing low quality cells, a principal component analysis was performed (Seurat::RunPCA). The PCs used was determined by an Elbow plot (Seurat::ElbowPlot). In this case, we decided to use the first 15 PCs for the following steps, including identify neighbors (Seurat::FindNeighbors), made UMAP projection (Seurat::RunUMAP). Finally, the clusters were identified by using Louvain clustering with resolution as 0.5 (Seurat::FindClusters). The UMAP projection and clustering information were extracted and imported into Loupe Browser for more customized visualization.

#### EdU and pimonidazole injections

In order to label mitotic cells with EdU, mice were injected intraperitoneally with thymidine analogue 5-Ethynyl-2′-deoxyuridine (EdU, 50 μg/g) (Sigma-Aldrich) 3 hours before sample collection. For labeling tissue hypoxia, pimonidazole (Hypoxyprobe) was prepared as 100 mg/ml in 0.9% saline, and was injected intraperitoneally (60 mg/kg) 1.5 to 2 hours before sample collection.

#### Tamoxifen treatment on mice

Mice expressing *Krt14-CreER*, as well as their wild-type controls, were treated with the topical application of 0.1% 4-Hydroxytamoxifen (4OH-Tam) diluted in 100% ethanol for 4 days, to manipulate the gene expression in the epidermis. After three days of resting period, the experiments were performed on the back skin of mice as indicated.

#### Doxycycline treatment on mice

Second telogen mice expressing *Krt14-rtTA*, as well as their control littermates, were put on a high-dose doxycycline (Dox, 2 mg/kg) food chow starting 2 days before the first punch biopsy. The mice were also injected intraperitoneally with 25 μg of Dox per gram of body weight at the time of first punch biopsy. For neonatal mice experiments, pregnant females were put on the high-dose Dox chow one day before they gave birth. Neonatal mice skins were harvested 48, 72, 96 hours after the start of doxy chow.

#### *In utero* lentiviral transduction

Concentrated lentiviral solutions were produced, and ultrasound-guided *in utero* injection of concentrated lentivirus was performed in the Comparative Biology Center at The Rockefeller University. Specifically, female mice were anesthetized with isoflurane at embryonic day 9.5, and 500 nL to 1 μL of lentivirus was injected into the amniotic sacs of the animal to selectively transduce individual progenitors within the surface ectoderm that will give rise to the skin epithelium.

#### Histology

Mouse back skin was dissected, and fixed with 4% paraformaldehyde diluted in PBS at 4°C overnight. After extensive washing with PBS, the tissues were incubated with 35% Ethanol for 1 hour and then 70% Ethanol for 1 hour. Samples in 70% Ethanol were then sent to Histowiz for processing as well as H&E and Trichrome staining.

#### Toluidine blue staining and TEM

Skin samples were fixed in 2% glutaraldehyde, 4% paraformaldehyde, and 2 mM CaCl_2_ in 0.1 M sodium cacodylate buffer (pH 7.2) for >1 hour at room temperature, post-fixed in 1% osmium tetroxide, and processed for Epon embedding; ultrathin sections (60–65 nm) were counterstained with uranyl acetate and lead citrate. Images were acquired with a transmission electron microscope (TEM, Tecnai G2–12; FEI, Hillsboro, OR) equipped with a digital camera (AMT BioSprint29). Semithin sections (800 nm) were stained with toluidine blue and photographed with a Zeiss Axio Scope equipped with a Nikon Digital Sight camera.

#### Immunoblot analysis

Cells were lysed in chilled 1x RIPA buffer (10x stock, EMD Millipore) diluted in PBS containing 1 tablet of cOmplete EDTA free pro- tease inhibitor and PhosSTOP phosphatase inhibitor for 30 minutes on ice. Protein was quantified using a Pierce BCA protein quantification kit. 20 μg of total protein lysates were loaded and separated on NuPAGE 4–12% Bis-Tris gels (Thermo Scientific). Proteins were transferred to nitrocellulose membranes, blocked for 1 hour with 5% milk in TBS-T, and incubated with the indicated primary antibodies diluted in TBS-T at 4°C overnight. Membranes were washed in TBS-T and incubated in HRP-coupled secondary anti- bodies at room temperature. Proteins were detected by chemiluminescence using ECL (Thermo Scientific) in a Bio-Rad ChemiDoc Imager. The following primary antibodies and dilutions were used: vinculin (Sigma, V9131 1:2000), HIF-1α (Cayman Chemical, 10006421, 1:1000), STAT3 (124H6, Cell Signaling, 1:1000), p-STAT3 (D3A7, Cell Signaling 1:1000), LDHA (21799–1-AP, Proteintech Group, 1:5000) and GLUT1 (ab115730 Abcam 1:1000). Western blot images were processed using Adobe Photoshop CS5.

#### ATAC-Seq library preparation and sequencing

ATAC-seq was performed on 70,000 FACS-purified cells from control and Day-1 wounded samples and processed as previously described.^58^ Briefly, cells were lysed in ATAC lysis buffer for 5 minutes and then transposed with TN5 transposase (Illumina) for 30 minutes at 37°C. Samples were uniquely barcoded, and the sequencing library was prepared according to manufacturer guidelines (Illumina). Libraries were sequenced on Illumina NextSeq 500. 40-bp paired-end ATAC-seq FASTQs were aligned to the mm10 genome from the Bsgenome.Mmusculus.UCSC.mm10 Bioconductor package (version 1.4.0) using Rsubread’s align method in paired-end mode with fragments between 1 to 5000 base-pairs considered properly paired.^98^ Normalized, fragment signal bigWigs were created.^99^ Peak calls for each replicate were made with MACS2 software in BAMPE mode.^76,100^

#### Cut and Run-Seq analysis

Cultured EpdSCs from GFPctrl_21%O2 (24hr) and IL22RA/IL20RB_1%O2 (24 hr) were trypsinized into single cell suspensions, and CUT&RUN was performed as previously described with minor modifications indicated below.^59^ Briefly, 650,000 cells were resuspended in crosslinking buffer (10 mM HEPES-NaOH pH 7.5, 100 mM NaCl, 1 mM EGTA, 1 mM EDTA, 1% formaldehyde) and rotated at room temperature for 10 minutes. Crosslinked cells were quenched with glycine at a final concentration of 0.125 M for 5 minutes at room temperature. Cells were washed with cold PBS and resuspended in NE1 buffer (20 mM HEPES-KOH pH7.9, 10 mM KCl, 1mM MgCl_2_, 1 mM DTT, 0.1% triton X-100 supplemented with Roche complete protease inhibitor EDTA-free) and rotated for 10 minutes at 4°C. Nuclei were washed twice with CUT&RUN wash buffer (20 mM HEPES pH7.5, 150 mM NaCl, 0.5% BSA, 0.5 mM spermidine supplemented with protease inhibitor) and incubated with concanavalin-A (ConA) beads washed with CUT&RUN binding buffer (20 mM HEPES-KOH pH 7.9, 10 mM KCl, 1 mM CaCl_2_, 1 mM MnCl_2_) for 10 minutes at 4°C. ConA-bead-bound nuclei were incubated CUT&RUN antibody buffer (CUT&RUN wash buffer supplemented with 0.1% triton X-100 and 2 mM EDTA) and antibody at 4°C overnight. After antibody incubation, ConA-bead-bound nuclei were washed once with CUT&RUN triton wash buffer (CUT&RUN wash buffer supplemented with 0.1% triton X-100) then resuspended and incubated at 4°C for 1 hour in CUT&RUN antibody buffer and 2.5 μL pAG-MNase (EpiCypher). ConA-bound-nuclei were then washed twice with CUT&RUN triton wash buffer, resuspended in 100μL of triton wash buffer, and incubated on ice for 5 minutes. Each 100 μl ConA-bound-nuclei was added with 2 μL 100 mM CaCl_2,_ mixed gently, and incubated on ice for 30 minutes. After adding 100 μL 2x stop buffer (30 mM EGTA), the reaction was incubated at 37°C for 10 minutes. After incubation, ConA-bound-nuclei were captured using a magnet, and the supernatant containing CUT&RUN DNA fragments was collected. The supernatant was incubated at 70°C for 2 hours with 2 μL 10% SDS and 2.5 μL 20mg/mL proteinase K. DNA was purified using PCI and overnight ethanol precipitation with glycogen at −20°C, and was resuspended in 15 μL of buffer EB. CUT&RUN sequencing libraries were generated using NEBNext Ultra II DNA Library Prep Kit for Illumina and NEBNext Multiplex Oligos for Illumina (Index Primer Set 1 and 2). PCR-amplified libraries were purified using 1.2x ratio of AMPure XP beads and eluted in 15 μL 0.1x TE buffer. All CUT&RUN libraries were sequenced on Illumina NextSeq using 40-bp paired-end reads. Reads were aligned to reference genome (mm10) using Bowtie2 (version 2.2.9) and deduplicated with Java (version 2.3.0) Picard tools (http://broadinstitute.github.io/picard). Reads were flittered to reads smaller or equal to 120 bp using samtools (version 1.3.1). BAM files for each replicate were combined using samtools. Bigwigs were generated using deeptools (version 3.1.2) with RPKM normalization and presented by Integrative Genomics Viewer (IGV) software. Peaks were called using SEACR using a stringent setting and a numeric threshold of 0.01. Peaks were further filtered to have peaks scores greater than 600 for a set of high confident peaks per antibody and condition. The motif analysis was performed with HOMER (version 4.10).

### QUANTIFICATION AND STATISTICAL ANALYSIS

Group sizes were determined on the basis of the results of the preliminary experiment and mice were assigned at random to groups. The number of animals shown in each figure is indicated in the legends as *n* = x mice per group and in times, and data are presented with multiple measurements per animal. Experiments were not performed in a blinded fashion. Statistical analysis was calculated using Prism software (GraphPad). All error bars are mean ± SEM. Experiments were independently replicated, and representative data are shown. Unpaired two-tailed Student’s t-tests were used to ascertain statistical significance between two groups, and one-way ANOVA was used to assess statistical significance between three or more groups with one experimental parameter; Two-way ANOVA was used to assess statistical significance between two or more groups with two experimental parameters. *, p < 0.05; **, p< 0.01; ***, p < 0.001; ****. p < 0.0001; ns, not significant. See figure legends for more information on statistical tests.

## Supplementary Material

MMC1

## Figures and Tables

**Table:** KEY RESOURCES TABLE

REAGENT or RESOURCE	SOURCE	IDENTIFIER
Antibodies		
Rabbit monoclonal anti-Phospho-STAT3 (Y705) antibody	Cell Signaling Technology	Cat# 9145S; RRID:AB_2491009
Rabbit monoclonal anti-HIF-1α antibody	Cell Signaling Technology	Cat# 36169; RRID:AB_2799095
Rabbit monoclonal anti-Glucose transporter GLUT1 antibody	Abcam	Cat# ab115730; RRID:AB_10903230
Rat monoclonal anti-CD31 antibody	Biolegend	Cat# 102502; RRID:AB_312909
Rat monoclonal anti-Endomucin antibody	Santa Cruz Biotechnology	Cat# sc-65495; RRID:AB_2100037
Chicken polyclonal anti-GFP antibody	Abcam	Cat# ab13970; RRID:AB_300798
Rat monoclonal anti-CD140a antibody	Biolegend	Cat# 135909; RRID:AB_2043973
Rat monoclonal anti-CD49e (Integrin-α5) antibody	Biolegend	Cat#103801; RRID:AB_31305
Chicken polyclonal anti-Keratin 14 antibody	Biolegend	Cat# 906004; RRID:AB_2616962
Rabbit polyclonal anti-Collagen I antibody	Abcam	Cat# 21286; RRID:AB_446161
Armenian Hamster monoclonal anti-PECAM-1 antibody	Millipore Sigma	Cat# MAB1398Z; RRID:AB_94207
Rabbit monoclonal anti-Ki-67 antibody	Cell Signaling Technology	Cat# 12202; RRID:AB_2620142
Goat polyclonal anti-Arginase 1 antibody	Novus Biologicals	Cat# NB100-59740; RRID:AB_892299
Polyclonal Goat anti-VEGFA antibody	R&D systems	Cat# AF-493-NA; RRID:AB_354506
Mouse monoclonal anti-Vinculin antibody	Millipore Sigma	Cat# V9131; RRID:AB_477629
Rabbit polyclonal anti-HIF-1α antibody	Cayman Chemical	Cat# 10006421; RRID:AB_409037
Mouse monoclonal anti-STAT3 antibody	Cell Signaling Technology	Cat# 9139; RRID:AB_331757
Rabbit polyclonal anti-LDHA antibody	Proteintech Group	Cat# 21799-1-AP; RRID:AB_10858925
Rat monoclonal PE anti-Integrin α6 antibody	eBioscience	Cat# 12-0495-82; RRID:AB_891474
Rat monoclonal anti-CD49f (Integrin α6) antibody	BD Pharmingen	Cat# 555734; RRID:AB_2296273
Rat monoclonal eFluor660 anti-CD34 antibody	eBioscience	Cat# 50-0341-82; RRID:AB_10596826
Rat monoclonal BV421 anti-CD34 antibody	BD Biosciences	Cat# 562608; AB_11154576
Rat monoclonal PerCP/Cy5.5 anti-Sca-1 antibody	Biolgened	Cat# 108124; RRID:AB_893615
Rat monoclonal APC/Cy7 anti-CD45 antibody	Biolegend	Cat# 103116; RRID:AB_312981
Rat monoclonal PE/Cy7 anti-CD31 antibody	Biolegend	Cat# 102524; RRID:AB_2572182
Rat monoclonal Biotin anti-CD117 antibody	Biolegend	Cat# 105804; RRID:AB_313213
Rat monoclonal BV421 anti-CD140a antibody	Biolegend	Cat# 135923; RRID:AB_2814036
Rat monoclonal APC anti-CD140a antibody	Biolegend	Cat# 135907; RRID:AB_2043969
Streptavidin PE-Cy7 conjugate	eBioscience	Cat# 25-4317-82; RRID:AB_10116480
Rat monoclonal BV421 anti-CD90.2 antibody	Biolegend	Cat# 140327; RRID:AB_2686992
Rat monoclonal BV421 anti-CD11b antibody	Biolegend	Cat# 101235; RRID:AB_10897942
Rat monoclonal BV421 anti-I-A/I-E (MHCII) antibody	Biolegend	Cat# 107621; RRID:AB_493726
Rat monoclonal AF488 anti-CD49e (Integrin α5) antibody	Biolegend	Cat# 103810; RRID:AB_528839
Rat monoclonal APC anti-CD49e (Integrin α5) antibody	Biolegend	Cat# 103813; RRID:AB_2750076
Rat monoclonal PE anti-Ly-6G antibody	Biolegend	Cat# 127607; RRID:AB_1186104
Rat monoclonal APC anti-Ly-6G antibody	Biolegend	Cat# 127613; RRID:AB_1877163
Rat monoclonal PE/Cy7 anti-CD117 (c-Kit) antibody	Biolegend	Cat# 105813; RRID:AB_313222
Rat monoclonal FITC anti-Ly-6C antibody	Biolegend	Cat# 128006; RRID:AB_1186135
Rat monoclonal APC anti-Siglec F antibody	Biolegend	Cat# 155508; RRID:AB_2750237
Armenian Hamster monoclonal PE/Cy7 anti-FcεRIα antibody	eBioscience	Cat# 25-5898-82; RRID:AB_2573493
Mouse monoclonal BV605 anti-CD64 antibody	Biolegend	Cat# 139323; RRID:AB_2629778
Rat monoclonal anti-CD16/CD32 antibody	eBioscience	Cat# 14-0161-85; RRID:AB_467134
Donkey polyclonal AF488 anti-Rabbit IgG antibody	Jackson ImmunoResearch Laboratories	Cat# 711-545-152; RRID:AB_2313584
Donkey polyclonal AF488 anti-Chicken IgG antibody	Jackson ImmunoResearch Laboratories	Cat# 703-545-155; RRID:AB_2340375
Donkey polyclonal AF488 anti-Rat IgG antibody	Jackson ImmunoResearch Laboratories	Cat# 712-545-150; RRID:AB_2340683
Goat polyclonal AF488 anti-Armenian hamster IgG antibody	Jackson ImmunoResearch Laboratories	Cat# 127-545-099; RRID:AB_2338996
Donkey polyclonal AF546 anti-Rabbit IgG antibody	Jackson ImmunoResearch Laboratories	Cat# 711-165-152; RRID:AB_2307443
Donkey polyclonal RRX anti-Rat IgG antibody	Jackson ImmunoResearch Laboratories	Cat# 712-295-150; RRID:AB_2340675
Donkey polyclonal AF647 anti-Rabbit IgG antibody	Jackson ImmunoResearch Laboratories	Cat# 711-605-152; RRID:AB_2492288
Donkey polyclonal AF647 anti-Rat IgG antibody	Jackson ImmunoResearch Laboratories	Cat# 712-605-150; RRID:AB_2340693
Donkey polyclonal HRP anti-Rabbit IgG antibody	Jackson ImmunoResearch Laboratories	Cat# 711-035-152; RRID:AB_10015282
Donkey polyclonal HRP anti-Mouse IgG antibody	Jackson ImmunoResearch Laboratories	Cat# 715-035-150; RRID:AB_2340770
Bacterial and virus strains		
NEB^®^ Stable Competent E. coli (High Efficiency)	New England Biolabs	Cat# C3040H
Chemicals, peptides, and recombinant proteins		
Normal Donkey Serum	Jackson ImmunoResearch Laboratories	Cat# 017-000-121; RRID:AB_2337258
Normal Goat serum	Jackson ImmunoResearch Laboratories	Cat# 005-000-121; RRID:AB_2336990
ProLong^™^ Diamond Antifade Mountant with DAPI	Thermo Fisher Scientific	Cat# P36962
Trypsin-EDTA (0.25%), phenol red	Gibco	Cat# 25200056
Liberase TL Research Grade	Sigma-Aldrich	Cat# 5401020001
ACK lysing buffer	Thermo Fisher Scientific	Cat# A1049201
T4 DNA ligase reaction buffer	New England Biolabs	Cat# B0202S
Nuclease-free water	Invitrogen	Cat# AM9937
RNaseOUT	Invitrogen	Cat# 10-777-019
NxGen phi29 DNA Polymerase	Lucigen	Cat# 30221-1-LU
Power SYBR^™^ Green PCR Master Mix	Thermo Fisher Scientific	Cat# 4367659
5-Ethynyl-2’-deoxyuridine (EdU)	Millipore Sigma	Cat# 900584
RIPA Lysis and Extraction Buffer	Thermo Fisher Scientific	Cat# 89901
cOmplete^™^ Protease Inhibitor Cocktail	Millipore Sigma	Cat# 11836145001
PhosSTOP^™^	Millipore Sigma	Cat# 4906837001
Illumina Tagment DNA Enzyme and Buffer Small Kit	Illumina	Cat# 20034197
NuPAGE^™^ LDS Sample Buffer (4X)	Thermo Fisher Scientific	Cat# NP0008
4’6’-diamidino-2-phenylindole (DAPI)	Millipore Sigma	Cat# 28718-90-3
Blasticidin	InvivoGen	Cat# ant-bl-05
Alt-R^®^ CRISPR-Cas9 tracrRNA, 20 nmol	IDT	Cat# 1072533
Alt-R^™^ S.p. Cas9 Nuclease V3, 100 μg	IDT	Cat# 1081058
TRI Reagent	Millipore Sigma	Cat# T3934
Complete mouse endothelial cell medium kit	Cell Biologics	Cat# M1168
Hypoxyprobe Kit (100 mg pimonidazole HCl plus 1.0 ml of 4.3.11.3 mouse MAb)	Hypoxyprobe	Cat# HP1-100Kit
Precision Plus Protein^™^ Dual Color Standards	Biorad	Cat# 1610374EDU
NuPAGE^™^ 4 to 12%, Bis-Tris, 1.0-1.5 mm	Thermo Fisher Scientific	Cat# NPG321BOX
NuPAGE^™^ MOPS SDS Running Buffer (20X)	Thermo Fisher Scientific	Cat# NPGGG1
NuPAGE^™^ Transfer Buffer (20X)	Thermo Fisher Scientific	Cat# NPGGG61
1x Tris Buffered Saline (TBS)	Biorad	Cat# 161G782
Pierce^™^ ECL Plus Western Blotting Substrate	Thermo Fisher Scientific	Cat# 32132
DNase 1 from bovine pancreas	Millipore Sigma	Cat# D4263
Human Plasma Fibronectin Purified Protein	Millipore Sigma	Cat# FCG1G
Corning Collagen I, Rat Tail	Corning	Cat# 354236
Poly-L-lysine	Millipore Sigma	Cat# P47G7
DreamTaq Green PCR Master Mix (2X)	Thermo Fisher Scientific	Cat# K1G82
Recombinant Mouse IL-24 (NS0-expressed) Protein (Carrier-free)	R&D Systems	Cat# 78G7-ML-G1G/CF
Recombinant Mouse IL-17A Protein (Carrier Free)	R&D Systems	Cat# 421-ML-G1G/CF
Doxycycline hydrochloride	Millipore Sigma	Cat# D3447
(Z)-4-Hydroxytamoxifen	Millipore Sigma	Cat# H79G4
Critical commercial assays		
RNase-Free DNase Set	Qiagen	Cat# 79254
TruSeq RNA Library Preparation Kit	Illumina	Cat# RS-122-2GG1
NEBNext Ultra II DNA Library Prep kit for Illumina	New England BioLabs	Cat# E7645L
NEBNext^®^ Multiplex Oligos for Illumina^®^ (Index Primers Set 1)	New England BioLabs	Cat# E7335S
NEBNext^®^ Multiplex Oligos for Illumina^®^ (Index Primers Set 2)	New England BioLabs	Cat# E75GGS
Agencourt AMPure XP beads	Beckman Coulter	A6388G
Direct-zol RNA Microprep	Zymo Research	Cat# R2G62
Direct-zol RNA Miniprep	Zymo Research	Cat# R2G5G
SuperScript^™^ VILO^™^ cDNA Synthesis Kit	ThermoFisher	Cat# 11754G5G
Power SYBR^™^ Green PCR Master Mix	ThermoFisher	Cat# 4368577
Pierce^™^ BCA Protein Assay Kit	ThermoFisher	Cat# 23225
LIVE/DEAD Fixable Blue Dead Cell Stain Kit	Thermo Fisher Scientific	Cat# L231G5
Click-iT^™^ EdU Cell Proliferation Kit for Imaging, Alexa Fluor^™^ 647 dye	Thermo Fisher Scientific	Cat#C1G34G
Click-iT^™^ EdU Cell Proliferation Kit for Imaging, Alexa Fluor^™^ 594 dye	Thermo Fisher Scientific	Cat# C1G339
Deposited data		
Raw bulk RNA-sequencing data	This paper	GEO: PRJNA7313G4
Raw 10x single cell RNA-sequencing data	This paper	GEO: PRJNA885G18
Raw ATAC-sequencing and Cut-and-Run sequencing data	This paper	GEO: PRJNA731164
Experimental models: Cell lines		
C57BL/6 mouse primary dermal microvascular endothelial cells	Cell Biologics	Cat# C57-6G64
293TN Producer Cell Line	System Biosciences	Cat# LV9GGA-1
Primary mouse fibroblasts	Fuchs Lab	N/A
Primary mouse keratinocytes	Fuchs Lab	N/A
J2 fibroblast feeder cells	Fuchs Lab	N/A
Experimental models: Organisms/strains		
Mouse: C57BL/6J	The Jackson Laboratory	Cat# GGG664; RRID:IMSR_JAX:GGG664
Mouse: Rosa26-stop-lox-stop YFP: B6.129X1-Gt(ROSA)26Sor^tm1(EYFP)Cos^/J	The Jackson Laboratory	Cat# GG6148; RRID:IMSR_JAX:GG6148
Mouse: Il24^−/−^	Fuchs Lab	N/A
Mouse: Il2Grb^−/−^	Genentech	N/A
Mouse: ${\text{Hif1α}}^{\text{fl/fl}}$: B6.129-Hif1a^tm3Rsjo^/J	The Jackson Laboratory	Cat# 7561; RRID:IMSR_JAX:GG7561
Mouse: K14CreER: K14-CreER-Rosa26-YFP	Fuchs Lab	N/A
Mouse: Glut1^fl/fl^: Slc2a1tm1.1S^tma^/AbelJ	The Jackson Laboratory	Cat# 31871; RRID:IMSR_JAX:031871
Mouse: Stat3^fl/fl^: B6.129S1-Stat3^tm1Xyfu^/J	The Jackson Laboratory	Cat# 16923; RRID:IMSR_JAX:016923
Mouse: Myd88^−/−^: B6.129P2(SJL)-Myd88^tm1.1Defr^/J	The Jackson Laboratory	Cat# 9088; RRID:IMSR_JAX:009088
Mouse: Trif^−/−^: C57BL/6J-Ticam1^Lps2^/J	The Jackson Laboratory	Cat# 005037; RRID:IMSR_JAX:005037
Mouse: Rag2^−/−^Il2rg^−/−^: C57BL/6NTac.Cg-Rag2^tm1Fwa^ Il2rg^tm1Wjl^	Taconic	Cat# 4111-F
Mouse: C57BL/6NTac	Taconic	Cat# B6-F
Mouse: TNFR1/TNFR2 DKO: B6.129S-Tnfrsf1b^tm1Imx^ Tnfrsf1a^tm1Imx^/J	The Jackson Laboratory	Cat# 003243; RRID:IMSR_JAX:003243
Mouse: Krt14-rtTA	Fuchs Lab	N/A
Mouse: Krt14-rtTA; sleeping beauty shIl24	Fuchs Lab	N/A
Oligonucleotides		
Quantitative real-time PCR primers (see Table S4)	Eurofins Genomics	N/A
Recombinant DNA		
pTY-EF1A-puroR-2a	Liu et al.^62^	A gift from Zhijian Chen
pTY-EF1A-HygromycinR-2a	Liu et al.^62^	A gift from Zhijian Chen
lentiCRISPRv2 blast	Stringer et al.^67^	Addgene plasmid #98293; RRID:Addgene_98293
pT4/HB	Wang et al.^68^	Addgene plasmid #108352; RRID:Addgene_108352
pCMV(CAT)T7-SB100	Má té s et al.^69^	Addgene plasmid #34879; RRID:Addgene_34879
pMD2.G	A gift from Didier Trono	Addgene plasmid #12259; RRID:Addgene_12259
psPAX2	A gift from Didier Trono	Addgene plasmid #12260; RRID:Addgene_12260
Software and algorithms		
Prism	https://www.graphpad.com/scientific-software/prism/	N/A
ImageJ	Schneider et al.^70^	https://imagej.nih.gov/ij/
FlowJo	https://www.flowjo.com	N/A
Adobe Photoshop	Adobe.com	N/A
Adobe Illustrator CS5	Adobe.com	N/A
R	R Development Core Team^71^	http://www.r-project.org/
TxDb.Mmusculus.UCSC.mm10.knownGene (R package)	Team BC and Maintainer BP^72^	https://bioconductor.org/packages/release/data/annotation/html/TxDb.Mmusculus.UCSC.mm10.knownGene.html
Salmon (version 1.4.0)	Patro et al.^73^	https://github.com/COMBINE-lab/salmon/releases
tximport (R package)	Soneson et al.^74^	https://bioconductor.org/packages/release/bioc/html/tximport.html
Bsgenome.Mmusculus.UCSC.mm10 (version 1.4.0) (R package)	Team TBD^75^	https://bioconductor.org/packages/release/data/annotation/html/BSgenome.Mmusculus.UCSC.mm10.html
MACS2 software in BAMPE mode	Zhang et al.^76^	https://pypi.org/project/MACS2/
Bowtie2 (version 2.2.9)	Langmead and Salzberg^77^	https://sourceforge.net/projects/bowtie-bio/files/bowtie2/2.2.9/
Java (version 2.3.0)	http://www.java.com	N/A
SAM tools (version 1.3.1)	Li et al.^78^	https://sourceforge.net/projects/samtools/files/samtools/1.3.1/
deeptools (version 3.1.2)	Ramıŕez et al.^79^	https://pypi.org/project/deepTools/
Integrative Genomics Viewer (IGV) software	Robinson et al.^80^	https://software.broadinstitute.org/software/igv/
HOMER (version 4.10)	Heinz et al.^81^	http://homer.ucsd.edu/homer/
BD FACSDiva software	BD Biosciences	N/A
Zen software	Carl Zeiss	N/A
Other		
Axio Observer Z1 epifluorescence microscope	Carl Zeiss	N/A
BioTek Cytation 5 Cell Imaging Multimode Reader	Agilent	N/A
2100 Bioanalyzer Instrument	Agilent	N/A
7900HT Fast Real-Time PCR system	Applied Biosystems	N/A
BD FACSAria Cell Sorter	BD Bioscience	N/A
BD LSRII Analyzer	BD Bioscience	N/A
BD LSRFortessa Analyzer	BD Bioscience	N/A
ChemiDoc Imager	Bio-Rad	N/A
2900 Biochemistry Analyzer	YSI	N/A
Sterile 4 mm biopsy punch	Integra Miltex	Cat# 33-34 SH
Sterile 6 mm biopsy punch	Integra Miltex	Cat# 33-36 SH

## Highlights

- Upon injury, IL-24 is induced specifically in epithelial stem cells at wound edges
- *Il24* is regulated by hypoxia and STAT3, independent of microbes, B cells, or T cells
- IL-24 acts in autocrine and paracrine signaling to regulate proliferation and metabolism
- Epithelial stem cells sense tissue damage and orchestrate organ-level repair

## INCLUSION AND DIVERSITY

We worked to ensure diversity in experimental samples through the selection of the cell lines. One or more of the authors of this paper self-identifies as a member of the LGBTQIA+ community. While citing references scientifically relevant for this work, we also actively worked to promote gender balance in our reference list. We avoided “helicopter science” practices by including the participating local contributors from the region where we conducted the research as authors on the paper.
