# Supplementary material for: A tissue injury sensing and repair pathway distinct from host pathogen defense
Source: Cell. Author manuscript; Available in PMC 2023 Jul 5. (PMC10321318; doi:10.1016/j.cell.2023.03.031)
Supplement: MMC1 [file NIHMS1894855-supplement-MMC1.pdf]

Cell, Volume *186*

Supplemental information

A tissue injury sensing and repair pathway distinct from host pathogen defense

Siqi Liu, Yun Ha Hur, Xin Cai, Qian Cong, Yihao Yang, Chiwei Xu, Angelina M. Bilate, Kevin Andrew Uy Gonzales, S. Martina Parigi, Christopher J. Cowley, Brian Hurwitz, Ji-Dung Luo, Tiffany Tseng, Shiri Gur-Cohen, Megan Sribour, Tatiana Omelchenko, John Levorse, Hilda Amalia Pasolli, Craig B. Thompson, Daniel Mucida, and Elaine Fuchs

Table S1 A list of cytokines known to activate STAT3, Related to Figure 1.

Cytokines	Corresponding Receptors
**IL6 family:** CLCF1, IL6, IL11, IL12, LIF, OSM	CNTFR, IL6RA/IL6ST, IL11RA/IL6ST, IL12RB1/IL12RB2, LIFR, LIFR/IL6ST, OSMR/IL31RA
**IL6 family:** CTF1, CNTF, GCSF, IL31, Leptin (LEP), IL27	CLC/CNTFR, CNTFR, GCSFR/CD114, IL31RA, LEPR/IL6RA, IL27RA
**IL10 family:** IL19, IL20, IL22, IL24, IL10	IL20RA/IL20R2, IL20R1/IL20R2 and IL22R/IL20R2, IL22R/IL10R2, IL20R1/IL20R2 and IL22R/IL20R2, IL10R1/IL10R2
**IFN family:** IFNA, IFNB, IFNG	IFNAR1/IFNAR2, IFNAR1/IFNAR2, IFNGR1/IFNGR2
**RTK family:** EGF, TGFA, amphiregulin (AREG), HBEGF, epiregulin (EREG), and betacellulin (BTC)	EGFR
**RTK family:** HGF	cMet
**RTK family:** PDGF, CSF-1	PDGFR1/PDGFR2, CSF1R

Table S2 IL24 protein sequence alignment across mammals, Related to Figure 1.

```
CLUSTAL O(1.2.4) multiple sequence alignment


House_mouse           --------------------------MSWGLQILPCLSLILLLWNQVPGLEGQEFRFGSC      34
Rat                   ------------------------MQTSLRQQILPGLSLILLVLNQVPELQGQEFRFGPC      36
Human                 -MNFQQRLQSLWTLARPFCPPLLATASQMQMVVLPCLGFTLLLWSQVSGAQGQEFHFGPC      59
Chimpanzee            WVRGPASQAASLSFSRPFCPPLLATASQMQMAVLPCLGFTLLLWSQVSGAQGQEFQFGPC      60
Black_flying_fox      ------------------------MGSPMQKAALPCLSFILLVWSWAPGVQGQEFQFGSC      36
Horse                 ------------------------MGSPMQRAALLSLSLILLLWSQRPGVQGQEFQFGSC      36
Dog                   -MN---------------ALWASSSRSTWGFLVMPCLSLILLLRSQGPGVQGQEFRFGPC      44
Pig                   ------------------------MGSPAPRAALPCLGLILLLWSQGPGVQGQEFQFGPC      36
Giant_Panda           -MVGKGR-----------ETRDQRMGFPGQTTALLCLSLILLLWSQAPGVQGQEFQFGPC      48
Polar_bear            ------------------------MGFSRQTTALLCLNLILLLWSQAPGVQGQEFQFGPC      36
Sperm_whale           ------------------------MGSPVHMSTLPCLSLILLFWSQGPGVQGQEFQFGPC      36
Killer_whale          ------------------------MGSPVRMAALPCLSLILLLWSQGPGVQGQEFQFGPC      36
Dolphin               ------------------------MGSPVRMAALPCLSLILLLWSQGPGVQGQEFQFGPC      36
                                                       :  *.: **. .     :****:** *

House_mouse           QVTGVVLPELWEAFWTVKNTVQTQDDITSIRLLKPQVLRNVSGAESCYLAHSLLKFYLNT      94
Rat                   QVTGVVLPELWEAFWTVKNTVKTQDELTSVRLLKPQVLQNVSDAESCYLAHSLLKFYLNT      96
Human                 QVKGVVPQKLWEAFWAVKDTMQAQDNITSARLLQQEVLQNVSDAESCYLVHTLLEFYLKT      119
Chimpanzee            QVKGVVPQKLWEAFWAVKDTTQAQDNITSARLLQQEVLQNVSDAESCYLVHTLLEFYLKT      120
Black_flying_fox      RVKGVDFQELWEAFQAMKDIVQAQDNITSIRLLRREVLQNVSDTESCYLIRALLKFYLNT      96
Horse                 RVEGVVLQELWAAFRAVKDVVQAQDNITGVRLLRKEVLQNVSDAESCYLSQALLKFYLDT      96
Dog                   RVQGVALRELREAFWTVKDTVQAKDNITSVRLLRKEVLQDVSDAESCYLIRALLKFYLNT      104
Pig                   RVEGIVLQELWEAFWDMKDVVQAQDNITNVRLLRKEVLQNVSEAESCYLIHSLLKFYLNT      96
Giant_Panda           RVEGVVLQELWEAFWAMKDIVQAKDNITSVRLLRKEVVQNVSGAESCYLIHALLKFYLNT      108
Polar_bear            RVEGVVLQELWEAFSAMKDIVQAKDNITSVRLLRKEVLQNVSDAESCYLIRALLKFYLTT      96
Sperm_whale           QVEGVVLQELWEAFQAMKDIAQAQDNITSVQLLRKEVLQNVSEAESCYLIHALLEFYLNT      96
Killer_whale          QVEGVVLQELWEAFQAMKDIVQAQDNITSVQLLRKEVLQNVSEAESCYLIHALLEFYLNT      96
Dolphin               QVEGVVLQELWEAFQAMKDIVQAQDNITSVQLLRKEVLQNVSEAESCYLIHALLEFYLNT      96
                      :* *:   :*  **  :*:  :::*::*. :**: :*:::** :***** ::**:*** *

House_mouse           VFKNYHSKIAKFKVLRSFSTLANNFIVIMSQLQPSKDNSMLPISESAHQRFLLFRRAFKQ      154
Rat                   VFKNYHSKIVKFKVLKSFSTLANNFLVIMSKLQPSKDNAMLPISDSARRRFLLYHRTFKQ      156
Human                 VFKNYHNRTVEVRTLKSFSTLANNFVLIVSQLQPSQENEMFSIRDSAHRRFLLFRRAFKQ      179
Chimpanzee            VFKNYHNRTVEVRTLKSFSTLANNFVLIVSQLQPSQENEMFSIRDSAHRRFLLFRRAFKQ      180
Black_flying_fox      VFKSYHEKAAEFRILKSFSTLANNFIFIASKLQPSVSRTKLGPGESARRRFLLFQRAFKQ      156
Horse                 VFKNYHGKAAEFRILKSFSTLANNFIAITSRLRPSQENEMFSISESARRRFLLFQREFKQ      156
Dog                   VFKNYLDEAADVRIRRSFSTLANNFFVIASKLQPSQEDEMFSISESARRRFLLFQRAFKQ      164
Pig                   IFKNYREKAVKFRILRSFSTLANNFVVIMSKLQPSQENEMFPISENARRRFLLFQREFKQ      156
Giant_Panda           VFKNYLDKAADSRIRKSFSTLANNFFVIVSKLQPSKENEMLSISESARRRFLLFQRAFKQ      168
Polar_bear            VFKNYLDKAADSRIRKSFSTLANNFFVIVSKLQPSQENEMFSISESARRRFLLFQRAFKQ      156
Sperm_whale           VFKNYHDKAVEFRILKSFSTLANNFIVIMSKLQPSQEKEMFSIRESARRRFLLFRRAFKQ      156
Killer_whale          VFKNYRDKAVEFGILKSFSTLANNFIVIVSKLQPSQEKEMFSISESAHRRFLLFQRAFKQ      156
Dolphin               VFKNYHDKAVEFRILKSFSTLANNFIVIVSKLQPSQEKEMFSISESARRRFLLFQRAFKQ      156
                      :**.*  . ..    :*********. * *:*:** .   :   :.*::****::* ***

House_mouse           LDTEVALVKAFGEVDILLTWMQKFYHL       181
Rat                   LDIEVALAKAFGEVDILLAWMQNFYQL       183
Human                 LDVEAALTKALGEVDILLTWMQKFYKL       206
Chimpanzee            LDVEAALTKALGEVDILLTWMQKFYKL       207
Black_flying_fox      LDIEAAQTKAFGEVDILLTWMQKFYQL       183
Horse                 LDIEAALTKAFGEVDILLTWMEKFYQP       183
Dog                   LDIQAAQTKAFGEVDILLTWMEKFYEF       191
Pig                   LDREVALTKAFGEMDILLTWMETFYQR       183
Giant_Panda           LDIQAAQTKAFGEVDILLTWMEKFYQF       195
Polar_bear            LDIQAAQTKAFGEVDILLTWMEKFYQF       183
Sperm_whale           LDREAAVTKAFGEVDILLTWMENFYH-       182
Killer_whale          LDREAAVTKAFGEVDILLTWMENFYQV       183
Dolphin               LDREAAVTKAFGEVDILLTWMEKFYQI       183
                      ** :.* .**:**:****:**:.**.
```

Table S3 A list of human cytokines and their receptors for homology analysis, Related to Figure 1.

Ligand	Annotation	Receptor subunit A	Receptor subunit B	Ligand	Annotation	Receptor subunit A	Receptor subunit B	Additional Receptor subunit
CLCF1	Cardiotrophin-like cytokine factor 1	CNTFR		IFNL3	Interferon lambda-3	IL10RB	IFNLR1	
CNTF	Ciliary neurotrophic factor	CNTFR		IFNL4	Interferon lambda-4	IL10RB	IFNLR1	
CSF1	Macrophage colony-stimulating factor 1	CSF1R		IFNW1	Interferon omega-1	IFNAR1	IFNAR2	
CSF2	Granulocyte-macrophage colony-stimulating factor	CSF2RA	CSF2RB	IL10	Interleukin-10	IL10RA	IL10RB	
CSF3	Granulocyte colony-stimulating factor	CSF3R		IL11	Interleukin-11	IL11RA	IL6ST	
CSH1	Chorionic somatomammotropin hormone 1	PRLR		IL12A	Interleukin-12 subunit alpha	IL12RB1	IL12RB2	
CSH2	Chorionic somatomammotropin hormone 2	PRLR		IL13	Interleukin-13	IL13RA	IL2RG	
CSHL1	Chorionic somatomammotropin hormone-like 1	PRLR		IL15	Interleukin-15	IL15RA	IL2RG	
EPO	Erythropoietin	EPOR		IL19	Interleukin-19	IL20RA	IL20RB	
FLT3LG	Fms-related tyrosine kinase 3 ligand	FLT3		IL2	Interleukin-2	IL2RA	IL2RB	IL2RG
GH1	Somatotropin	GHR		IL20	Interleukin-20	IL22RA1	IL20RB	IL20RA
GH2	Growth hormone variant	GHR		IL21	Interleukin-21	IL21R	IL2RG	
IFNA1	Interferon alpha-1/13	IFNAR1	IFNAR2	IL22	Interleukin-22	IL22RA1	IL20RB	
IFNA10	Interferon alpha-10	IFNAR1	IFNAR2	IL23A	Interleukin-23 subunit alpha	IL12RB1	IL23R	
IFNA14	Interferon alpha-14	IFNAR1	IFNAR2	IL24	Interleukin-24	IL22ra1	IL20rb	IL20RA
IFNA16	Interferon alpha-16	IFNAR1	IFNAR2	IL26	Interleukin-26	IL20ra	IL10rb	
IFNA17	Interferon alpha-17	IFNAR1	IFNAR2	IL3	Interleukin-3	IL3RA	CSF2RB	
IFNA2	Interferon alpha-2	IFNAR1	IFNAR2	IL34	Interleukin-34	CSF1R		
IFNA21	Interferon alpha-21	IFNAR1	IFNAR2	IL4	Interleukin-4	IL4RA	IL2RG	
IFNA4	Interferon alpha-4	IFNAR1	IFNAR2	IL5	Interleukin-5	IL5RA	CSF2RB	
IFNA5	Interferon alpha-5	IFNAR1	IFNAR2	IL6	Interleukin-6	IL6RA	IL6ST	
IFNA6	Interferon alpha-6	IFNAR1	IFNAR2	IL7	Interleukin-7	IL7R		
IFNA7	Interferon alpha-7	IFNAR1	IFNAR2	KITLG	Kit ligand	KIT		
IFNA8	Interferon alpha-8	IFNAR1	IFNAR2	LEP	Leptin	LEPR		
IFNB	Interferon beta	IFNAR1	IFNAR2	LIF	Leukemia inhibitory factor	LIFR	IL6ST	
IFNE	Interferon epsilon	IFNAR1	IFNAR2	OSM	Oncostatin-M	OSMR	IL31RA	
IFNG	Interferon gamma	IFNGR1	IFNGR2	PRL	Prolactin	PRLR		
IFNK	Interferon kappa	IFNAR1	IFNAR2	THPO	Thrombopoietin	MPL		
IFNL1	Interferon lambda-1	IL10RB	IFNLR1	TSLP	Thymic stromal lymphopoietin	IL7R	CRLF2	
IFNL2	Interferon lambda-2	IL10RB	IFNLR1					

Table S4 A list of gene-specific qPCR primers used in the study, Related to STAR Methods.

1	*mHprt_F*	GATCAGTCAACGGGGGACATAAA	36	*mTgfa_R*	CAAGCAGTCCTTCCCTTCAG
2	*mHprt_R*	CTTGCGCTCATCTTAGGCTTTGT	37	*mAreg_F*	GACTCACAGCGAGGATGACA
3	*mIl6_F*	TCCATCCAGTTGCCTTCTTG	38	*mAreg_R*	CTGTGATAACGATGCCGATG
4	*mIl6_R*	GGTCTGTTGGGAGTGGTATC	39	*mEreg_F*	TCTGACATGGACGGCTACTG
5	*mIfnb_F*	CCCTATGGAGATGACGGAGA	40	*mEreg_R*	CGCAACGTATTCTTTGCTCA
6	*mIfnb_R*	CTGTCTGCTGGTGGAGTTCA	41	*mBtc_F*	GCACAGGTACCACCCCTAGA
7	*proIl1bF*	GGGCCTCAAAGGAAAGAATC	42	*mBtc_R*	GCCCCAAAGTAGCCTTTCTC
8	*proIl1bR*	TACCAGTTGGGGAACTCTGC	43	*mIl22ra1F*	AGG TCC ATT CAG ATG CTGGT
9	*mIfnaF*	ATTTTGGATTCCCCTTGGAG	44	*mIl22ra1R*	TAG GTG TGG TTG ACG TGGAG
10	*mIfnaR*	TATGTCCTCACAGCCAGCAG	45	*mIl20Ra_F*	GAAGAACGTGGTCCCAGTGT
11	*mIl24_F*	GCCCAGTAAGGACAATTCCA	46	*mIl20Ra_R*	AAGTAGCCAATTGCGGAGAA
12	*mIl24_R*	ATTTCTGCATCCAGGTCAGG	47	*Eef1a1_F*	AACCACCGCTAATTCAAAGCAA
13	*mIl22_F*	CCGAGGAGTCAGTGCTAAGG	48	*E ef1a1_R*	AGGAGCCCTTTCCCATCTCAG
14	*mIl22_R*	CATGTAGGGCTGGAACCTGT	49	*mIl20rb_F*	CCTCCCAGACACCTTGAAAA
15	*mEgf_F*	GGGAAAATGTGTCTCCCTCA	50	*mIl20rb_R*	CAAAGAGATGCTCCGAGGAC
16	*mEgf_R*	TCATGCCTGACACCATGATT	51	*mPpib_F*	TGATCCAGGGTGGAGACTTC
17	*mHbegf_F*	CAGGACTTGGAAGGGACAGA	52	*mPpib_R*	ATTGGTGTCTTTGCCTGCAT
18	*mHbegf_R*	CCGTGGATGCAGTAGTCCTT	53	*mIfng_F*	GCGTCATTGAATCACACCTG
19	*mIl11_F*	CATTGGGATCTTTGCAGCTT	54	*mIfng_R*	TGAGCTCATTGAATGCTTGG
20	*mIl11_R*	GAGCTGTAAACGGCGGAGTA	55	*mPdk1_F*	GTGCCCCTGGCTGGGTTTGG
21	*mLif_F*	AGAAGGTCCTGAACCCCACT	56	*mPdk1_R*	CCAGGCGTCCCATGTGCGTT
22	*mLif_R*	CCACACGGTACTTGTTGCAC	57	*mVegfa_F*	GGAGAGCAGAAGTCCCATGA
23	*mClcf1_F*	CGAGCCTGACTTCAATCCTC	58	*mVegfa_R*	ACTCCAGGGCTTCATCGTTA
24	*mClcf1_R*	TACGTCGGAGTTCAGCTGTG	59	*mPgk1_F*	ATTCTGCTTGGACAATGGAGC
25	*mHgf_F*	ATGGGGAATGAGAAATGCAG	60	*mPgk1_R*	AGGCATGGGAACACCATCA
26	*mHgf_R*	CTCCCTCACATGGTCCTGAT	61	*mIl24_3UTRF1*	GTTGTTGGCTCAGGCTTTTC
27	*mIl12a_F*	CATCGATGAGCTGATGCAGT	62	*mIl24_3UTRR1*	GTTTCCAGGGAAGGTGACAA
28	*mIl12a_R*	CAGATAGCCCATCACCCTGT	63	*mIl24_456F*	CACTCTGGCCAACAACTTCA
29	*mOsm_F*	TCAGGGGTCTGATGACACAA	64	*mIl24_608R*	GCTTTCACCAAAGCGACTTC
30	*mOsm_R*	GTGTGAGGTCACCCAGAGGT	65	*mIL17A_107F*	CAAACACTGAGGCCAAGGAC
31	*mIl19_F*	ATCCTGTCCCTGGAGAACCT	66	*mIL17A_324R*	CTTTCCCTCCGCATTGACAC
32	*mIl19_R*	AAAGAGTTGGCAATGCTGCT	67	*mGlut1_F*	TGCAGCCCAAGGATCTCTCT
33	*mIl20_F*	TCTACCAGACCCCTGACCAC	68	*mGlut1_R*	CGGCTTGCCCGAGATCT
34	*mIl20_R*	CATTGCTTCTTCCCCACAAT	69	*mIL22_240F*	CCGAGGAGTCAGTGCTAAGG
35	*mTgfa_F*	ATCACCTGTGTGCTGATCCA	70	*mIL22_345R*	CATGTAGGGCTGGAACCTGT

Supplemental Table 1. A list of cytokines known to activate STAT3, Related to Figure 1.

Cytokines that are known to activate STAT3 are shown and are grouped into cytokine families (first column). Corresponding cytokine receptors are shown in the second column. The cytokine receptors that have a TPM value >=1 is considered to be expressed in EpdSCs based on RNA-seq. The cytokines (highlighted in red) whose receptors are expressed in epidermal stem cells were examined for their expression in the micro-dissected wounded skin.

Supplemental Table 2. IL24 protein sequence alignment across mammals, Related to Figure 1.

IL24 protein sequences from different mammals were obtained from NCBI and aligned using Clustal Omega. Asterisk (*) indicates positions that have a single, fully conserved residue; Colon (:) indicates conservation between groups of strongly similar properties; Period (.) indicates conservation between groups of weakly similar properties.

Supplemental Table 3. A list of human cytokines and their receptors for homology analysis, Related to Figure 1.

A total of 59 human proteins (columns 1 and 2) that share sequence and structure homology with IL24 were bioinformatically extracted from human genome. These proteins were then subjected to initial homology alignment to generate a preliminary tree. Based on this tree, the representative cytokines from each clade/close family members highlighted in yellow, are then subjected to the same method to generate a smaller tree shown in Figure S1A. The receptors for the 59 human cytokines are listed (Columns 3-5). The receptors whose extracellular domain share homology with the IL24-receptors and contain >=2 tandem Ig-like domain were subjected to homology alignment and shown in Figure S1B.

Supplemental Table 4. A list of gene-specific qPCR primers used in the study, Related to STAR Methods.
